# Cultural adaptations to augment health and mental health services: a systematic review

**DOI:** 10.1186/s12913-016-1953-x

**Published:** 2017-01-05

**Authors:** Priscilla Healey, Megan L. Stager, Kyler Woodmass, Alan J. Dettlaff, Andrew Vergara, Robert Janke, Susan J. Wells

**Affiliations:** 1 Centre for the Study of Services to Children and Families, University of British Columbia, ASC 453, 3187 University Way, Kelowna, BC V1V 1V7 Canada; 2 University of Houston Graduate College of Social Work, 3511 Cullen Blvd Room 110HA, Houston, TX 77204-4013 USA; 3 University of British Columbia, Okanagan Campus Library, LIB 241, 3287 University Way, Kelowna, BC V1V 1V7 Canada

**Keywords:** Cultural safety, Cultural appropriateness, Cultural competence, Health, Mental health, Racial disparities, Ethnicity

## Abstract

**Background:**

Membership in diverse racial, ethnic, and cultural groups is often associated with inequitable health and mental health outcomes for diverse populations. Yet, little is known about how cultural adaptations of standard services affect health and mental health outcomes for service recipients. This systematic review identified extant themes in the research regarding cultural adaptations across a broad range of health and mental health services and synthesized the most rigorous experimental research available to isolate and evaluate potential efficacy gains of cultural adaptations to service delivery.

**Methods:**

MEDLINE, PsycINFO, CINAHL, EMBASE, and grey literature sources were searched for English-language studies published between January 1955 and January 2015. Cultural adaptations to any aspect of a service delivery were considered. Outcomes of interest included changes in service provider behavior or changes in the behavioral, medical, or self-reported experience of recipients.

**Results:**

Thirty-one studies met the inclusion criteria. The most frequently tested adaptation occurred in preventive services and consisted of modifying the content of materials or services delivered. None of the included studies focused on making changes in the provider’s behavior. Many different populations were studied but most research was concerned with the experiences and outcomes of African Americans. Seventeen of the 31 retained studies observed at least one significant effect in favor of a culturally adapted service. However there were also findings that favored the control group or showed no difference. Researchers did not find consistent evidence supporting implementation of any specific type of adaptation nor increased efficacy with any particular cultural group.

**Conclusions:**

Conceptual frameworks to classify cultural adaptations and their resultant health/mental health outcomes were developed and applied in a variety of ways. This review synthesizes the most rigorous research in the field and identifies implications for policy, practice, and research, including individualization, cost considerations, and patient or client satisfaction, among others.

**Electronic supplementary material:**

The online version of this article (doi:10.1186/s12913-016-1953-x) contains supplementary material, which is available to authorized users.

## Background

Many people of diverse racial, cultural, and ethnic groups in the United States and Canada experience differences in the quality of health and mental health services associated with their group identity [1, 2]. Differences in treatment occur in access to care [3, 4], quality of provider-patient interactions [5, 6], and engagement in care [7]. For example, in the U.S., a nationally-representative sample of Latinos was less likely than non-Latin whites to seek health information or refer to it in conversation with their physicians [8]. In California and Chicago studies, African Americans were less likely than whites to feel that they had received high-quality care or had their medical needs met [5, 9]. In Canada, Van Herk, Smith, and Andrew found Aboriginal mothers in an urban center felt disrespected by mainstream care providers [10]. Such differences have been associated with negative health and mental health outcomes including, for example, underuse of services [11, 12] and failure to comply with physician advice [13].

Health and mental health professionals are increasingly concerned with delivering more linguistically appropriate, culturally competent, and culturally safe services [14–16]. Systematic reviews imply that culturally-adapted interventions can be successful but the nature and process of the adaptations are often lost during the reporting of results [14, 17, 18] and many reviews are narrowly focused. Examples of the latter include diabetes care [19, 20], asthma [21], HIV [22, 23], obesity [24], and psychotherapy [25, 26]. Critical assessment of the literature is complicated by the nature of the research conducted. For example, Lie, Lee-Rey, Gomez, Bereknyei, and Braddock reviewed the efficacy of cultural adaptations for service providers but found no studies with equivalent control groups [27]. To demonstrate efficacy, cultural adaptations must be compared to the same intervention, minus the adaptations in question. Unless the experimental and comparison groups are identical (but for the adaptation), it is impossible to determine whether any observed effect resulted from the adaptation itself, or some other aspect of the intervention. No systematic reviews have yet aggregated studies from the health and mental health literature which isolate cultural adaptations from other aspects of the intervention and/or research design (see Additional file 1 for a list of reviews that are related to this study’s research question).

Research questions were developed in consultation with an advisory panel of experts, knowledge users, and community representatives. The researchers sought evidence of cultural adaptations to any aspect of service delivery which impact: (a) the behavior of the service provider, (b) the recipient’s self-reported experience, or (c) outcomes for the service recipient. Distinctions among the terms, race, ethnicity, and culture are essential to understanding these issues. Markus and Moya describe race as group membership assigned to people based on “perceived physical and behavioral human characteristics” and used as a basis for the conferral of “differential … power, and privilege” [28]. Ethnicity is described as “ideas and practices” through which people identify with a group based, for example, on “commonalities including … language, history, nation, …customs, …and/or ancestry”, and culture as “ideas and practices attached to all the important social distinctions in our lives” [28]. Cultural competence, appropriateness, and safety each have specific implications for improved service delivery. See Additional file 2 for more detailed definitions. For the purpose of this article, the term cultural adaptation is used to represent all modifications made to standard service methods in order to make services more acceptable, relevant, useful, and/or effective for diverse populations. The terms patient, client, and consumer are used interchangeably depending on the context of the references cited.

## Methods

The project methodology is consistent with the Cochrane Collaboration guidelines and supplemental sources [29–34]. Due to the vast quantity of information available on this topic and a burgeoning interest in the field, it was necessary to adopt stringent criteria with regard to inclusion in this review. The scope of this review was progressively narrowed to include only randomized controlled trials (RCTs) and quasi-experimental research with parallel cohorts. Only studies which isolated the cultural adaptation from control interventions were considered. This limited the kinds of adaptations which could be included in the study. For example, changes in organizational policy were of interest to the project, but no studies met the strict criteria for comparison groups. The search included adaptations in any aspect of service delivery. Outcomes of interest included: (1) health outcomes of the recipient, (2) behavioral outcomes of the recipient, (3) self-reported outcomes of the recipient, including service satisfaction, and (4) behavioral outcomes of the service provider. A complete summary of reviewers’ inclusion and exclusion criteria is provided in Table 1.

**Table 1:** Reviewers’ inclusion and exclusion criteria

Inclusion	Exclusion
1. English language from any country	1. Study findings not in English
2. Published 1950 or after	2. Prior to 1950 or abstracts not available
3. RCTs and quasi-experimental designs with parallel cohorts of control or comparison groups	3. Studies which were not RCTs or quasi-experimental designs, e.g., observational studies, moderator analyses
4. Services included health or mental health	4. Other human services
5. Described adaptation(s) intended to make services more responsive to or effective for diverse racial and ethnic populations; adaptations may target: • individual service provider OR • service system	5. Did not contain a description of the specific activities undertaken to improve cultural competence, appropriateness, or safety, and/or the study did not justify the inclusion of an adaptation with culturally-grounded rationale and/or existing research
6. Explicitly tested the effectiveness of the cultural adaptation separate from any other health or mental health intervention studied. This must result in intervention and control groups that differ only on the included cultural adaptation	6. Studies in which the cultural component and the health or mental health intervention were not evaluated separately from the other service provided. Also excluded studies that tested a generally used intervention to study its impact on a cultural, minority, ethnic, or disadvantaged population without adapting it to specifically suit the needs of the target population
7. Focus of study was on provision of a service	7. Studies that: only tested the translation of psychometric instruments, questionnaires, and diagnostic tools, focused on engaging visible minorities in research, or involved service delivery at some unspecified future time, such as genetic registries
8. Studies pertained to people and organizations in the mainstream culture making adjustments to include and serve those who are subject to inequity in service delivery or service outcomes	8. Service recipients did not represent a group subject to disparities in service delivery or outcomes, or target subjects’ data were confounded with those of another group that is not subject to health disparities and/or is not the target of the cultural adaptation under study
9. Reported outcomes that included: • change in service provider behavior OR • change in self-reported experience or outcomes of service recipient OR • change in observed outcomes for service recipient	9. Did not contain evidence of having measured outcomes of the adaptation to enhance cultural competence, appropriateness, or safety with specific reference to: • change in service provider behavior OR • change in self-reported experience or outcomes of service recipient OR • change in observed outcomes for service recipient
10. Outcomes and data were provided and analyzed in a way that allowed an evaluation of the direct results of the cultural adaptation	10. Outcomes and conclusions were not substantiated in the report with sufficient data
11. There were no flaws in the study methodology and/or delivery deemed likely to threaten the internal validity and interpretability of the study’s results	11. The research design, intervention delivery, or assessment of outcomes involved a confounding variable which threatens the internal validity of results, e.g., clinically meaningful differences between groups at baseline, lack of experimental control, inadequate statistical reporting, etc.

### Search strategy

The final strategy was iterative; the search results guided refinement of the search terms. The original database search of MEDLINE, PsycINFO, CINAHL, and EMBASE was performed in August and September 2011. A combination of keywords and database-specific subject headings were used to search the following concepts and synonyms: “cultural competency” or “culturally tailored” or “racial disparities” or intercultural or “communication barriers” related to race or ethnicity. See Additional file 3 for a complete list of search terms, dates the searches were conducted, as well as full database search histories. The results were updated in 2012 and again in 2015. In addition to the database search, key reports and literature reviews were identified and hand-searched. These documents were selected based on the degree to which they focused on the project’s research questions. A forward citation title search was conducted using Google Scholar and Web of Knowledge; items found were screened by title and abstract. Backward citation searches involved title-screening the reference lists of key reports and literature reviews to identify any relevant literature cited within this study’s search findings. Twenty percent of these results were double-screened by a second reviewer to ensure consistency. Authors of retained reports were also contacted to identify research that may have been missed in the search. A grey literature search was conducted to identify unpublished or omitted material (see Additional file 4). Inclusion of a database was guided by relevance to the study focus and relevance of returns from initial searches.

### Screening

Titles and abstracts were used to eliminate documents that were deemed irrelevant or outside the scope of the research questions. Reviewers then evaluated the full-text of documents and applied the inclusion criteria to identify the strongest research in the health and mental health literature. At the onset of the study, four pairs of reviewers conducted title and abstract screening. Each pair independently double-screened a sample of the same documents and established inter-rater reliability using Cohen’s Kappa. Reviewers discussed any disagreements to resolve them, and consulted the Principal Investigator (PI) where an agreement could not be reached. Consistency among reviewers was maintained through use of the same decision rules, constant communication, meetings, and oversight from the PI. Once reviewers achieved a Kappa of .90, each reviewer screened items independently. Reliability was periodically checked by double-rating a random 10% sample of the screened articles for each set of at least 100 reviewed documents, then producing a new Kappa. When the Kappa slipped below .90, reviewers returned to double-screening each document until an agreement of .90 or greater was achieved. In the 2015 update, sufficient resources were available to double-screen all database documents.

In full-text screening, each pair of raters double-screened until they achieved 100% agreement on a random sample of documents, at which point they worked independently, double-screening a random 20% to assess reliability. Because inter-rater agreement remained at or near 100%, double-screening was reduced to a random 10% of every 100 documents. In addition, any article included at this stage was cross-screened by the second reviewer to confirm the validity of the inclusion decision. In the event of a disagreement, the PI was consulted to settle the discrepancy. During the 2015 update, resources were again available for double-screening of all documents. Authors were contacted for additional information when necessary.

### Data extraction

The data extraction form was based on the work of Hasnain et al. [18], Littell et al. [32], and the Cochrane Collaborative GRADE approach [35]. The form included, but was not limited to: details of the study population, baseline characteristics, details of the setting, study methodology, study outcomes, and bias/quality information. Inter-rater reliability was assessed by comparing the content of extraction forms until 100% agreement was attained. In 2012, a random third of articles was compared for consistency. This was reduced to a random 10% because reviewers maintained consistent inter-rater reliability.

The 2015 data extraction began with a trial period in which three reports were extracted and evaluated in consultation with the PI to ensure raters’ accuracy and comprehension of the process. The remaining reports were double-extracted independently by each member of a single pair of reviewers. Reports were discussed in-depth with the PI when: 1) the reviewers disagreed with one another, or 2) the reviewers’ decisions changed as a result of discussion and consideration of the research design. Some reports presented issues which necessitated further specification of the inclusion/exclusion criteria. For example, Breitkopf et al. studied culturally-framed messages for African American, Latina, and White women, but collapsed data across these three ethnicities, confounding the populations of interest with White data [36]. This resulted in a need to specify that data for the population of interest must be evaluable in isolation from the general population. The final inclusion process was iterative. Articles from all three stages of the project were revisited and discussed until the current pool of items was identified.

## Results

### Search results

**Fig. 1:**
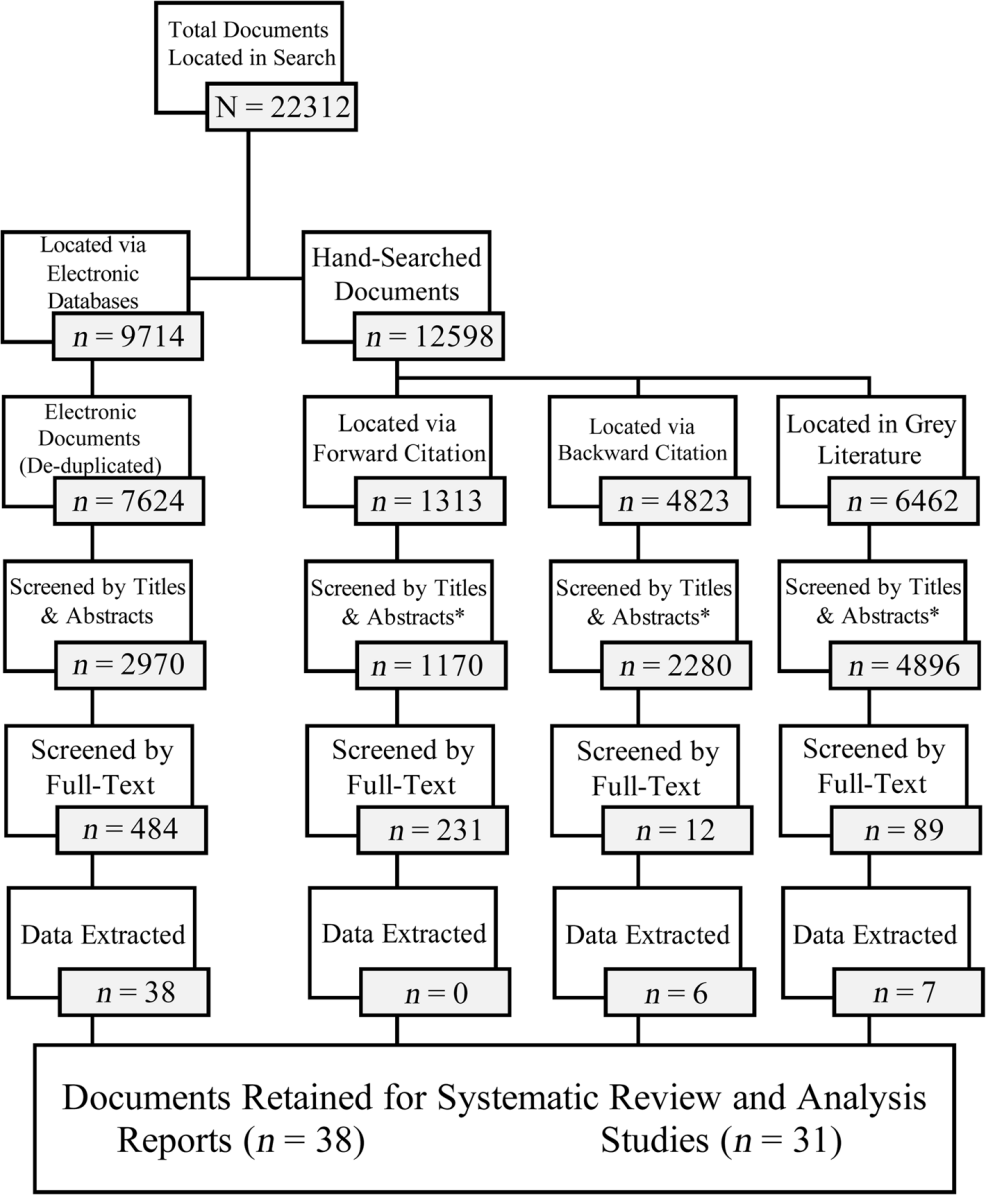
Breakdown of results during the search process. *Documents have been de-duplicated where possible given database and software restrictions

The electronic databases returned the following results: 2011 (*n* = 5141), 2012 update (*n* = 529), and 2015 update (*n* = 1954) after de-duplication, for a total number of electronic database documents of 7624. The flow of documents retained at each step in the review process is charted in Fig. 1.

For the reports reviewed during title and abstract screening, the most common reason for elimination was that they did not test a culturally-adapted intervention to improve health or mental health outcomes for service recipients. The remaining eliminations were studies of translated instruments or studies lacking comparison/control groups with parallel cohorts. In full-text screening, the vast majority were omitted because the interventions in the control or comparison groups were not identical, save for the cultural adaptation in question. One study was omitted at the point of data extraction because the results reported in narrative could not be substantiated by the data provided in the publication. A handful of reports were omitted because they could not be obtained from the authors. Requests made to key authors produced no additional studies or findings.

### Study characteristics

The search identified 38 retained documents published in the United States from 1993 to 2015. Three documents represented completed pilots (two of which were subsequently folded into the analysis of the final report), and five represented interim findings that could be combined with later final results, yielding 31 studies. The research represented a total of 9,831 participants. The majority of studies targeted a specific racial, ethnic, or cultural group: African American (*n* = 17), Latino (*n =* 7), Asian American (*n* = 4), and Gay/Bisexual (*n* = 1). Two studies targeted ethnically-diverse populations [37, 38]. All 31 studies tested a culturally-adapted intervention for the service recipients. Topics were diverse and included enhancement of diet through increased fruit and vegetable intake, cessation of smoking, uptake of preventive services, and improvement in psychological functioning. In total, 17 of the 31 studies observed significant effects in favor of a culturally adapted intervention.

#### Risk of bias

Each study was assessed by two reviewers for bias in seven areas outlined by the GRADE Criteria: selection, allocation concealment, performance, detection, attrition, reporting, and other. Authors were contacted for further design details where possible. Of the retained studies, only one reported enough information to fully assess bias in each domain. Thirteen studies were rated *high* risk for bias in one or more domains, and 30 studies had an *unknown* risk of bias in one or more area. Table 2 provides a summary of reviewers’ bias ratings.

**Table 2:** Reviewers’ judgments regarding sources of bias

Study	Selection bias	Allocation concealment	Performance bias	Detection bias	Attrition bias	Reporting bias	Other bias
Ard et al. 2008 [53]	X	X	?	?	√	?	?
Burrow-Sanchez et al. 2015 [77]	√	?	X	?	√	?	X
Burrow-Sanchez & Wrona, 2012 [76]	√	?	X	?	√	?	?
Chiang & Sun, 2009 [79]	X	?	?	?	√	?	?
Fitzgibbon et al. 2005 [50]	X	X	√	√	√	√	?
Gondolf, 2008 [47]	√	?	?	?	√	√	√
Halbert et al. 2010 [68]	?	?	?	?	√	√	√
Havranek et al. 2012 [58]	√	√	√	√	?	?	?
Holt et al. 2009 [71]	?	?	?	?	√	?	?
Holt et al. 2012a [66]; 2012b [67]	√	?	√	√	√	?	X
Huey & Pan, 2006 [64]; Pan et al. 2011 [65]	?	?	?	√	√	√	?
Hwang et al. 2015 [80]	√	?	?	√	√	?	X
Jandorf et al. 2013a [51]; 2013b [52]	√	?	?	?	√	?	√
Johnson et al. 2005 [37]	?	?	?	?	√	√	√
Kalichman et al. 1993 [46]	?	?	?	?	?	?	?
Kreuter et al. 2004 [60]; 2005 [62]	√	√	?	√	√	√	√
La Roche et al. 2006 [38]	√	?	X	?	√	?	X
Lee et al. 2013 [54]	√	?	?	?	?	?	?
McCabe et al. 2009 [55]; 2012 [56]	√	√	X	√	√	?	√
Mohan et al. 2014 [59]	√	√	X	√	√	?	√
Newton & Perri, 2004 [45]	?	?	?	?	√	√	√
Nollen et al. 2007 [73]	√	√	√	√	√	√	√
Orleans et al. 1998 [74]	X	?	?	X	√	X	√
Resnicow et al. 2009 [63]	?	?	√	?	√	√	√
Sanders Thompson et al., 2010 [70]	√	?	?	?	?	?	?
Shoptaw et al. 2003 [78]	√	?	?	?	√	√	√
Skaer et al. 1996 [57]	X	?	?	√	√	?	?
Unger et al. 2013 [72]	√	?	?	√	√	?	X
Wang et al. 2012a [48]; 2012b [49]	√	?	?	?	√	?	√
Webb, 2009 [75]	?	?	√	√	√	√	√
Webb et al. 2010 [69]	?	?	?	?	√	?	?
Total Low Risk: Total Unclear Risk: Total High Risk:	√ = 17 ? = 9 X = 5	√ = 5 ? = 24 X = 2	√ = 6 ? = 20 X = 5	√ = 18 ? = 12 X = 1	√ = 27 ? = 4 X = 0	√ = 11 ? = 19 X = 1	√ = 14 ? = 12 X = 5

### Analysis of cultural adaptations

The included studies used a range of cultural adaptations designed to promote cultural competence, appropriateness, or safety. A variety of frameworks for classification of the results were reviewed, but none were directly applicable to the assortment of adaptations found. For example, Chowdhary et al. [39] elaborated on the targets of adaptation in psychotherapy as well as on how to develop adaptations. They described adaptations of language, therapist adaptations such as matching or training, use of metaphors to increase cultural relevance, adapting the content of therapy, adapting communication to incorporate cultural constructs such as somatization, client-derived treatment goals, changes in therapeutic methods, and addressing clients’ socio-economic contexts to reduce barriers to treatment. While helpful in characterizing the psychological treatments reviewed, the framework does not incorporate the breadth of health and mental health adaptations found in this review. Castro, Barrera, and colleagues’ [40, 41] reviews report similar findings. In 2004, they outlined the sources of mismatch between the provider and consumer (group characteristics, program delivery staff, and administrative/community factors) that could be used as targets of intervention [41]. In 2006 they presented a Heuristic Framework [42] that may be used to guide the development of adaptations. In 2012, they identified several frameworks for characterizing adaptations, most notably, Bernal et al.’s eight dimensions of therapy that could be culturally adapted [41]. The elements included aspects of treatment such as goals, methods, and context of treatment, as well as characteristics of the client that could be incorporated such as language and familiar cultural expressions [43].

Although these frameworks include many aspects of health and mental health services, the conceptualization is somewhat abstract and the categories have not yet been developed to the extent that they could be used as a more specific classification scheme. For instance, a local healer could be classified as therapist matching, inclusion of cultural knowledge, or modification of treatment methods. The work done to date is helpful, but not yet sufficient for classification of wide-ranging adaptive cultural arrangements and activities.

To establish a framework better suited for the purposes of this review, the two senior researchers in the study conducted a content analysis of the research included to identify three primary domains of adaptation: 1) community outreach and involvement, 2) changes in the structure and process of service delivery, and 3) adaptation of content. Once the framework had been identified, it was expanded to encompass other adaptations encountered during the literature review that were deemed to be hypothetically testable in comparative research designs (Table 3). The adaptations described in the framework focus on changes that affect, in some way, the interface with the client. For example, it may be possible to make beneficial changes in organizational management or legislation, but it is the resulting changes to the nature of service delivery that are categorized, rather than the way in which the adaptations are generated.

**Table 3:** Conceptual framework for cultural adaptations

1. Community outreach and involvement
a. Community needs assessment (e.g., outlining the issue from their perspective)
b. Involvement in development of the adaptation
c. Participation in the implementation/management/delivery of services
2. Changes in structure and process of service delivery
a. Change in geography/location (e.g., location of center, home vs. office visits, etc.)
b. Change made to the physical space (e.g., pictures, room or building design, etc.)
c. Change in mechanism of service delivery (e.g., face-to-face, electronic, mailed, etc.)
d. Changes to service provider/presenter (e.g., selection and training)
i. Language matching to client
ii. Race, gender, or cultural matching to client
e. Change in manner of service delivery (e.g., interaction style, proximity to client, active or passive speech, intonation, rapport building, self-presentation, group composition, etc.)
f. Provision of supplemental services, resources, or support
i. Supplemental providers (e.g., traditional healer, patient navigators)
ii. Funds for a specific service or resource
iii. Supplemental services (e.g., child care, transportation, paid leave from work)
iv. Translated materials (e.g., documents, signs, etc.)
iv. Other
3. Adaptation of content
a. Level of personal specificity
i. Individualized
ii. Targeted to subgroup
b. Inclusion of cultural content
i. Graphics
ii. Cultural allusions (affect-free content with which the recipient may personally identify)
iii. Culturally-relevant factual information
iv. Targets or references negative-valence beliefs, values, or experiences (e.g., fatalism, stigmatization)
v. Targets or references neutral or positive-valence beliefs, values, or experiences (e.g., familial involvement, time-orientation)

This framework is designed to serve the full range of health and mental health domains and organize adaptations in such a way as to be as exhaustive and mutually exclusive as possible. Culturally sensitive changes to a treatment may have more than one adaptation, but they all can be categorized using the framework in Table 3, allowing practitioners and researchers to begin to describe and compare adaptions using a common approach. The following description and the accompanying analysis of the adaptations found are representative of this classification system. Resulting categorizations and descriptions of implemented adaptations are provided in Additional file 5. For future analyses of adaptation, the authors have also provided an example of how the classification could be adapted to reflect the depth and/or detail of adaptation addressed by Resnicow et al. [44] in Additional file 6.

#### Overview of adaptations

Within the retained studies, the most popular method of adapting an intervention was to modify the content of materials or dialogue to include racial, ethnic, or cultural facts, values, imagery, or other cultural components. The next most common was to change the manner in which a service was delivered, including increases in time and attention paid to recipients, cultural matching of providers to clients, and provision of additional resources. Consultation with community was implemented to inform, and in concert with, changes to structure, process, and/or content of service delivery. The majority of adaptations were tested in one or more of the retained studies.

There were no discernible differences in adaptation selection or impact when it came to specific health problems; adaptations from each category identified in the framework were fairly evenly featured for each unique health concern, and cultural adaptation was not particularly effective for any one health concern. There were likewise no discernible differences in adaptation selection or impact when it came to targeting specific cultural groups.

#### Community outreach and involvement

Fourteen studies included consultation with members of, and experts from, the community of interest. All fourteen studies engaged community members in the development of the adaptation’s content via focus groups, but community outreach and involvement was not the primary adaptive goal of any study. Focus groups typically elicited cultural themes, values, and preferences from participants.

#### Changes in structure and process of service delivery

Twenty-one studies featured changes in the structure and/or process of service delivery. Only Newton and Perri implemented and isolated changes to the physical intervention space between conditions, with the adapted program being held at a site located in the African American community [45].

##### Changes to service provider

Six studies tested provider matching. These studies matched the providers’ or presenters’ race [45–49], gender [46], or language [48, 49] to those of the recipients in an effort to facilitate changes in behavior, such as screening uptake. Beyond racial matching, three studies selected providers/presenters who were a cultural or community match to service recipients in an effort to enhance their identification with their provider [47, 50–52].

##### Change in manner of service delivery

Sixteen studies changed the manner in which an intervention was delivered or portrayed. Studies employed a wide variety of techniques, and typically included more than one adaptation. For example, Jandorf and colleagues had peer navigators relate their personal experiences with colonoscopy and model effective coping skills [51, 52]. Alternatively, Ard et al. organized group interventions such that all participants were of the same race [53], and Lee et al. had therapists spend time building rapport with clients and emphasized collaboration [54].

##### Provision of supplemental services, resources, or support

Five studies provided recipients with supplementary resources to facilitate uptake or retention in a service. Resources included provision of translated and simplified materials [55, 56], a voucher to be redeemed for a free mammogram in an effort to increase breast cancer screening [57], provision of access to child-care and transportation, and accommodation for recipients’ work schedules [54]. Havranek et al. provided a supplemental values-affirmation exercise to clients prior to meeting with their general practitioner to enhance self-efficacy [58], and Mohan, Riley, Schmotzer, Boyington, and Kripalani provided clients with a simplified and illustrated medication management tool to facilitate understanding of pharmaceutical regimens [59].

#### Adaptation of content

Twenty-six studies adapted the content of the intervention to reflect the norms, values, and culture of the target population. Studies referred to culturally-salient statistics and historical events as motivating factors for change. Adaptations often included positive cultural values, beliefs, and norms to facilitate, enhance, or motivate change during the intervention. Negative cultural values and experiences were frequently referred to as motivating factors (e.g., African Americans’ history of oppression), or were targeted by the adaptation as a barrier to change (e.g., belief in fatalism). Changes were implemented in many mediums of program content. Four studies included individualized content for each recipient. Three tailored intervention content and provider delivery based on recipients’ level of acculturation [60–65]. The study undertaken by McCabe and colleagues personalized parent–child interaction therapy after a family-based needs assessment; changes addressed cultural beliefs about the causes of behavioral problems, familial roles, discipline, etc. [55, 56].

#### Packages of adaptations

Of 31 studies, only five tested a lone adaptation. Twenty-seven^1^ studies tested multiple cultural adaptations in concert, such that it is impossible to isolate the effect of any one change to the service. It is possible that any observed effect may have resulted from: 1) a single adaptation from amongst the many, 2) the sum of adaptations made together, or 3) an interaction among adaptations made. Two studies employed multiple comparison groups, allowing for the isolation of numerous adapted components as well as the complete package of adaptations [46, 47]. The number of adapted elements ranged from one to five, based on an adaptation of Hasnain and colleagues’ framework for identifying the number of adaptations present in a service [18]. Additional file 7 includes a Forest Plot that illustrates the effects of number of adaptations tested.

### Analysis of outcomes

The retained studies tested health outcomes from five of the six domains identified as potentially meeting this review’s criteria regarding health outcomes (see Table 4 for the categorization chart).

**Table 4:** Conceptual framework for health outcomes

1. Service Provider Behavioral Outcomes 2. Service Uptake: Completion/Participation 3. Service Recipient Awareness, Beliefs, Knowledge, and Attitudes 4. Service Recipient Behavioral Outcomes 5. Indicators of Health/Mental Health Status	

No retained studies assessed service provider behavioral outcomes. Havranek et al. assessed client-provider communication from the client and provider perspectives, but clients were the intended target of the intervention [58]. Overall, 19 studies assessed the awareness, knowledge, and/or attitudes of recipients. Twenty-three of the studies assessed service uptake in some form. Sixteen studies assessed service recipient behavioral outcomes. Ten studies measured indicators of health or mental health status. Seventeen studies observed health or mental health outcomes which significantly favored the culturally adapted group, but there was no clear pattern as to which outcomes were affected, or which adaptations were implemented. However, three studies observed results which significantly favored the standard group.

Interventions implemented within the retained studies were focused either on targeting ongoing health concerns (treatment services) or on prevention of future health problems (preventive services). The interventions implemented by Mohan et al. and Havranek et al. were the only retained studies to adapt a physiological treatment service [58, 59]. In contrast, preventive services targeted physical conditions, such as cancer, HIV, smoking, and asthma. Decisions to obtain medical screening were categorized as *preventive service uptake* outcomes. No retained studies assessed the medical decisions made based on screening results.

In addition to testing the health outcomes of interest to this review, retained studies also tested some outcomes not directly related to health experience, such as *recipient shared material with a friend*. These outcomes did not fit the target definition for health outcomes, so intervention impact on such outcomes is not discussed herein. Studies are tallied and categorized based on their primary research goal. See Table 5 for a detailed list of results, accompanying data, and comparison groups for each study. Additional file 5 categorizes outcomes by type of adaptation and outcome.

**Table 5:** Characteristics of included studies and reported between-groups outcomes

Study	Sample	Intervention	Outcome
Ard et al. 2008 [53]	African Americans *N =* 377	*Culturally Adapted (CA):* Racially matched participants in group weight-loss program. *Standard (STD):* Multicultural participant group.	○^a^ No significant difference in attendance (*p* = .09), change in weight (*p* = .97), fruit/vegetable intake (*p* = .60), fiber intake (*p* = .94) or fat intake at follow-up (*p* = .46). ○ No significant difference in percentage of recipients getting >180 min. physical activity per week at follow-up (*p* = .18).
Burrow-Sanchez et al. 2015 [77]	Latinos (Adolescents) *N* = 70^b^	*Culturally Adapted (CA)*: Culturally tailored cognitive behavioral therapy (CBT). *Standard (STD)*: Standard CBT.	○ No significant difference in reduction of past-90-day drug use (*p* = .66).
Burrow-Sanchez & Wrona, 2012 [76]	Latinos (Adolescents) *N* = 35	*Culturally Adapted (CA)*: Culturally tailored cognitive behavioral therapy (CBT). *Standard (STD)*: Standard CBT.	○ No significant difference in reduction of past-90-day drug use or program retention^†^. ● Parents in the CA condition were more satisfied with the program (*p* = .02). ○ No significant difference in adolescent satisfaction, (*p* = .09).
Chiang & Sun, 2009 [79]	Asian Americans(Chinese) *N* = 128	*Culturally Adapted (CA)*: 8-week culturally tailored walking program. *Standard (STD)*: Non-tailored program.	○ No significant difference in post-test blood pressure or walking endurance^†^.
Fitzgibbon et al. 2005 [50]	African Americans (Obese/over-weight, women) *N* = 59	*Culturally Adapted (CA):* Faith-based 12-week weight-loss program. *Standard (STD):* Weight-loss intervention with no active faith component.	○ No significant difference in program retention (>75% attendance)^†^. ○ No significant difference in energy expenditure at 12 weeks (*p* = .08). ○ No significant difference in dietary fat consumption at 12 weeks (*p* = .91). ○ No significant difference in 12-week weight change: Kg (*p* = .34), % (*p* = .41). ○ No significant difference in BMI^c^ change at 12 weeks (*p* = .37, *d* = 0.27). ○ No significant difference in either vigorous physical activity (*p* = .36) or moderate physical activity (*p* = .06) at 12 weeks.
Gondolf, 2008 [47]	African Americans (Men) *N* = 372	*Culturally Adapted (CA1):* 16-week racially-matched group counseling program with standard curriculum for domestic-violence offenders. *Culturally Adapted (CA2):* Racially-matched counsellor and culturally-targeted program curriculum. *Standard (STD):* Multicultural group with Caucasian counsellors and standard curriculum.	○ Program completion was comparable across groups^†^.
Halbert et al. 2010 [68]	African Americans (Women) *N* = 176	*Culturally Adapted (CA):* Culturally tailored genetic counseling. *Standard (STD):* Standard genetic counseling.	○ No significant difference in risk perception at follow-up (*LRT* = 0.07, *p* = .79). ○ No significant difference in counseling completion (*p* = .70). ○ Genetic screening uptake was comparable between groups^†^.
Havranek et al. 2012 [58]	African Americans *N* = 99	*Culturally Adapted (CA):* A values-affirmation exercise to reduce stereotype-threat and boost self-efficacy of clients during race-discordant client-provider communications. *Standard (STD):* Neutral comparison exercise.	● CA group provided and requested significantly more information about medical condition (*p* = .03), but not therapeutic regimen (*p* = .56), lifestyle (*p* = .42), or services (*p* = .70). ○ No significant difference in trust in provider (*p* = .55) or patient visit satisfaction (*p* = .32).
Holt et al. 2009 [71]	African Americans (Men) *N* = 49	*Culturally Adapted (CA):* Spiritually-based “Sunday-school” prostate cancer education session. *Standard (STD):* Non-spiritual prostate cancer educational session.	○ Groups were comparable in rating the acceptability/appropriateness of the intervention and in finding it helpful for making informed decisions^†^. ● CA group read significantly more of the materials (*p* < .01). ○ Difference in change in self-efficacy was not significant between groups for screening, decision making regarding prostate specific antigen, or decision making regarding digital rectal examination^†^. ○ Groups changed comparably in screening beliefs, knowledge (prostate cancer, screening controversy, relationship between screening and mortality), and barriers to screening decisions^†^.
Holt et al., 2012a [66], 2012b [67]	African Americans *N* = 285	*Culturally Adapted (CA):* Spiritually-themed colorectal cancer education session. *Standard (STD):* Non-spiritual colorectal cancer education session.	○ No significant difference in CRC^d^ knowledge at follow-up (*p* = .65 [2012a]). ● STD group self-reported significantly more FOBT^e^ within previous 12 months (*p* = .03 [2012b]). ○ No significant difference in follow-up self-report of lifetime FOBT (*p* = .55), flexible sigmoidoscopy (*p* = .52), colonoscopy (*p* = .55), or barium enemas (*p* = .32 [2012b]). ○ No significant difference in follow-up perceived CRC screening benefits (*p* = .16), FOBT benefits (*p* = .20), FOBT barriers (*p* = .33), colonoscopy benefits (*p* = .80), or colonoscopy barriers (*p* = .54 [2012b]).
Huey & Pan, 2006 [64]; Pan et al. 2011 [65]	Asian Americans *N* = 30	*Culturally Adapted (CA):* Culturally tailored single-session exposure treatment for phobias. *Standard (STD):* Standard one-session exposure treatment for phobias.	○ No significant differences in avoidance/anxiety, catastrophic thinking, general fear, or DSM-IV TR^f^ phobic symptoms at follow-up (2011)^†^. ○ CA group had significantly lower subjective distress ratings at one week, but not at 6 months (2011)^†^. ● No significant difference of clinician rating of fear at one week, but the CA group was rated as having significantly lower fear response at six months (2011)^†^.
Hwang et al. 2015 [80]	Asian Americans (Chinese) *N* = 50	*Culturally Adapted (CA):* Culturally tailored CBT for depression. *Standard (STD):* Standard CBT.	○ No significant difference in program retention^†^. ○ No significant difference in severity of depression by session 12^†^. ● Log-linear growth model revealed CA group observed significantly greater decrease in depression scores from baseline to session 12 despite baseline differences (*p* = .047).
Jandorf et al., 2013a [51], 2013b [52]	African Americans *N* = 304^g^	*Culturally Adapted (CA):* Peer-led patient navigation for African Americans referred for colonoscopy. *Standard (STD):* Physician-led patient navigation.	○ Groups were similar in rates of colonoscopy screening at follow-up (2013b)^†^. ○ No significant difference in trust in provider at follow-up (*p* = .56 [2013a]). ○ No significant difference in perceived message and source credibility (*p* = .97 [2013a]). ○ Groups were comparable in satisfaction (*p* = .07 [2013a])^†^.
Johnson et al. 2005 [37]	Multicultural (Children) *N* = 3157	*Culturally Adapted (CA):* 8-session, 50 min. multicultural anti-smoking curriculum. *Standard (STD):* Standard anti-smoking curriculum.	○ No significant differences in past-month smoking or lifetime ever-having-smoked by 8th grade^†^.
Kalichman et al. 1993 [46]	African Americans (Women) *N* = 106	*Culturally Adapted (CA1):* Culturally tailored content and behavior of presenters in an AIDS/HIV educational video. *Culturally Adapted (CA2):* Racial and gender matching of presenter to audience in an HIV/AIDS educational video. *Standard (STD):* Standard HIV/AIDS educational video with mixed-gender/race presenters.	● CA1 obtained significantly more HIV tests (*p* < .01). ● CA1 and CA2 together were significantly more likely to request condoms at post-test, (*p* < .001). ○ No significant differences in HIV/AIDS information seeking at post-test, condom purchasing, or attempting to use more condoms^†^. ○ No significant differences in HIV/AIDS knowledge and attitudes at post-test^†^. ● CA1 presenters were significantly more perceived as expressing concern (*p* < 0.01) than the other groups combined. ○ No significant differences in ratings of presenter expertise^†^.
Kreuter et al. 2003 [61], 2004 [60], 2005 [62]	African Americans (Women) *N* = 599^h^	*Culturally Adapted (CA):* Culturally & behaviorally tailored cancer education magazines to increase mammography/fruit & vegetable intake. *Standard (STD):* Magazines tailored on behavioral content alone.	○ CA group was not significantly more likely to have obtained a mammogram by 18 months than the STD group (2005)^†^. ○ Groups increased comparably in median fruit/vegetable servings (2005)^†^. ○ No significant difference in having received and read materials at 6 months (2004)^†^.
La Roche et al. 2006 [38]	African Americans, Latinos *N* = 22^i^	*Culturally Adapted (CA):* Allocentric family asthma-management program. *Standard (STD)*: Standard family asthma-management program.	● CA group reduced the number of emergency department visits in the 12 month follow up period by 50%^†^. ● CA group was significantly greater in parental asthma knowledge at 12 months (*p* < .05). ○ No significant differences in parental skills, child skills, or child knowledge at 12 months^†^.
Lee et al. 2013 [54]	Latinos *N* = 53^j^	*Culturally Adapted (CA):* Culturally tailored single-session motivational interviewing to reduce alcohol-induced behavioral problems. *Standard (STD):* Standard motivational interviewing.	○ No significant difference in treatment engagement^†^. ○ No significant difference in program satisfaction^†^. ○ Groups decreased comparably from baseline in past-month heavy drinking. The CA group observed a non-significant, but greater effect (*p* = .08, η^2^ = 0.10). ● CA group had greater decreases in alcohol-induced problem behavior scores on the DrInC^k^ Implusivity subscale, (*p* = .009, η^2^ = 0.14). The other DrInC subscales did not significantly differ between groups^†^.
McCabe & Yeh, 2009 [55]; McCabe et al. 2012 [56]	Latinos (Mexican American) *N* = 58	*Culturally Adapted (CA):* Culturally tailored Parent–child Interaction Therapy (PCIT) for families with children who have behavior problems. *Standard (STD):* Standard PCIT.	○ CA group showed greater improvement for all health outcomes, but differences were all non-significant between groups: ECBI^l^ Intensity Subscale (*p* = .77, *d* = .09), ECBI Problem Subscale (*p* = .34, *d* = .28), CBCL^m^ (*p* = .10, *d* = .36), ECI^n^ ODD^o^ symptoms (*p* = .13, *d* = .07), ECI CD^p^ symptoms (*p* = .12, *d* = .26), ECI ADHD^q^ symptoms (*p* = .18, *d* = .08), PSI^r^ (*p* = .53, *d* = 0.09), and PLOC^s^ (*p* = .10, *d* = .35 [2012])^†^. ○ CA group showed significantly greater improvement on the CBCL Internalizing subscale (*p* = .049), but this was no longer significant after a Bonferroni correction (2012). ○ Groups were comparable in treatment satisfaction and dropout (2009)^†^. ○ No significant differences in parent–child positive/negative interaction styles (do and don’t skills [2009])^†^. ○ No significant difference in positive parenting behavior scores at post-test (2009)^†^.
Mohan et al. 2014 [59]	Latinos *N* = 200	*Culturally Adapted (CA):* TAU^t^ plus a supplementary simplified and illustrated medication management tool. *Standard (STD)*: TAU.	● CA group had significantly greater knowledge and understanding of medication regimens at follow-up (*p* < .001). ○ No significant difference in self-reported medication adherence at follow-up^†^.
Newton & Perri, 2004 [45]	African Americans *N* = 42^u^	*Culturally Adapted (CA):* 10-session culturally tailored group-exercise program and written materials. *Standard (STD):* Standard program and materials.	○ No significant difference in completion of prescribed exercise (*p* = .39). ● CA group rated group leaders as showing significantly more appreciation (*p* = .03). ○ No significant difference in self-reported physical activity at post-test^†^. ○ Groups increased comparably in maximum oxygen capacity^†^. ○ There was no significant difference in self-efficacy at post-test^†^.
Nollen et al. 2007 [73]	African Americans *N* = 500	*Culturally Adapted (CA):* Culturally-tailored anti-smoking video and print guide. *Standard (STD):* Standard video and print guide.	● CA group used the guide significantly more (*p* = .03). ○ No significant difference in video usage (*p* = .37), perceived benefits of the guide in attempting to quit (*p* = .07), or of the video in attempting to quit (*p* = .32). ○ No significant difference in progression along the Stages of Change continuum in terms of readiness to quit by 6 months^†^. ○ No significant difference in 7-day abstinence at 6 months (*p* = .27). ○ No significant difference in change from baseline in the number of cigarettes smoked per day at 6 months (*p* = .61) or self-reported nicotine patch use (*p* = .75).
Orleans et al. 1998 [74]	African Americans *N* = 1422	*Culturally Adapted (CA):* Culturally targeted stop-smoking counseling session and written materials. *Standard (STD):* Standard counseling and materials.	○ No significant difference in self-reported reading of material or proportion of recipients who found the guide helpful at 6 months^†^. ● STD group rated the guide as significantly more suitable for other family members at 6 months (*p* = .01). ● CA group significantly reduced the number of cigarettes smoked (*p* = .002), was more likely to set a quit date (*p* = .001), and was more likely to switch to a lower-nicotine brand of cigarettes by 6 months (*p* = .001). ● CA group made significantly more quit attempts (*p* = .007), and used more pre-quitting strategies (*p* = .05) by 6 months. ○ No significant difference in self-reported week-long abstinence, progression along the Stages of Change continuum, or in smoking abstinence by 6 months^†^. ● CA group had a higher quit rate (*p* = .034), and were more advanced along the Stages of Change continuum (*p* = .035) at 12 months. ○ No significant difference in nicotine patch or gum use, or median number of quit attempts at 12 months^†^.
Resnicow et al. 2009 [63]	African Americans *N* = 560	*Culturally Adapted (CA):* Culturally tailored fruit & vegetable promotional materials. *Standard (STD):* Standard materials.	○ No significant difference in mean daily fruit/vegetable intake by 3 months (*p* = .13). ○ Groups were comparable in self-reported reading of most/all newsletters at 3 months^†^.
Sanders Thompson et al. 2010 [70]	African Americans *N* = 771	*Culturally Adapted (CA):* Culturally tailored colorectal cancer risk-reduction materials. *Standard (STD):* Standard materials.	○ No significant difference in affect, engagement, ease of understanding, cognitive processing, or intent to screen at 22 weeks^†^.
Shoptaw et al. 2005 [78]	Gay/Bisexuals (Men) *N* = 80^v^	*Culturally Adapted (CA):* Culturally tailored cognitive behavioral therapy. *Standard (STD):* Standard cognitive behavioral therapy.	○ No significant difference in program retention^†^. ○ CA group significantly reduced self-reported unsafe receptive anal intercourse during first 4 weeks of treatment. Differences between conditions were non-significant at 12 months^†^. ● CA group had significantly higher Treatment Effectiveness Scores for meth abstinence at end of treatment (*p* < .05). ○ No significant difference in percent of negative urine samples or reported days of past-month meth use^†^.
Skaer et al. 1996 [57]	Latinas (Low-income, Women) *N* = 80	*Culturally Adapted (CA):* Provision of voucher to redeem for one free mammogram. *Standard (STD):* No voucher provided.	● CA group was 47 times more likely to obtain a mammogram at follow-up, using logistic regression analysis (*p* = .0001).
Unger et al. 2013 [72]	Latinos *N* = 139	*Culturally Adapted (CA):* Illustrated fotonovela to increase depression knowledge and reduce stigma. *Standard (STD):* Standard depression pamphlet.	● CA group was significantly lower in antidepressant stigma (*p* < .05) and mental health care stigma (*p* = <.05) at post-test^w^. ● CA group was significantly higher in depression knowledge at post-test (*p* < .05). ○ No significant differences in self-efficacy to identify depression or willingness to seek help (*p* > .05) at post-test.
Wang et al. 2012a [48]; 2012b [49]	Asian Americans (Chinese) *N* = 442^x^	*Culturally Adapted (CA):* Culturally tailored, mailed mammography promotional video. *Standard (STD):* Standard mailed mammography promotional video.	○ Groups were comparable in increases in mammography from baseline (2012b)^†^. ○ No significant differences in intent to obtain mammogram at post-test (2012a)^†^. ○ No significant difference in cultural views of healthcare, knowledge, perceived risk, perceived benefits, or perceived barriers at post-test (2012a)^†^.
Webb, 2009 [75]	African Americans *N* = 261	*Culturally Adapted (CA):* Culturally targeted written materials for smoking cessation. *Standard (STD):* Standard materials.	● CA material was significantly more likely to capture attention, provide encouragement, and help in quitting^†^. ● STD material was seen as significantly more credible (*p* < .05). ○ No significant difference in booklet utilization (*p* = .09). ● CA group was significantly more satisfied with content (*p* = .03). ● STD group was 1.97 (95% CI [1.09, 3.55]) times more likely to make a quit attempt by follow-up (*p* = .03). ● STD group scored significantly higher on the Contemplation Ladder measure at follow-up (*p* = .01). ○ No significant difference in point prevalent abstinence or smoking reduction^†^.
Webb et al. 2010 [69]	African Americans *N* = 243	*Culturally Adapted (CA):* Culturally targeted written materials for smoking cessation and exercise. *Standard (STD):* Standard smoking and exercise materials.	● CA group was significantly higher in perception of personal risks of smoking at post-test (*p* = .02, η^2^ = 0.02). ● CA group was significantly higher in perception of culturally-specific risks of smoking at post-test (*p* = .04, η^2^ = 0.02). ● CA group was significantly higher in intentions to quit at post-test (*p* = .04, η^2^ = 0.02). ○ No significant difference in Contemplation Ladder scores at post-test^†^. ○ No significant difference in smoking knowledge at post-test^†^.

^a^○ Denotes a non-significant outcome. ● Denotes a significant outcome as defined by the original authors’ criteria. ^†^Denotes an outcome which is reported in the original document, but for which probability values were not provided

^b^ *N*’s represent the sample size analyzed in the final report. Note that interim reports may have analyzed data representing a different sample size from that of the final report, e.g., due to attrition

^c^Body Mass Index

^d^Colorectal Cancer (CRC)

^e^Fecal Occult Blood Test (FOBT)

^f^Diagnostic and Statistical Manual of Mental Disorders, 4th Edition Text Revision (DSM-IV TR)

^g^Note: This number represents the sample size of the CA and STD groups only, omitting the TAU sample, which was not of central interest to this review

^h^Note: This number represents the sample size of the CA and STD groups only, omitting the “culturally relevant tailoring” group, as neither BRT not CRT + BRT can serve as an adequate control to test this group

^i^This number represents the number of families participating, not the number of individuals

^j^This number represents the number of participants that were said to be randomized

^k^Drinkers’ Inventory of Consequences (DrInC)

^l^Eyberg Child Behavior Inventory (ECBI)

^m^Child Behavior Checklist (CBCL)

^n^Early Childhood Inventory (ECI)

^o^Oppositional Defiant Disorder (ODD)

^p^Conduct Disorder (CD)

^q^Attention Deficit Hyperactivity Disorder (ADHD)

^r^Parenting Stress Index (PSI)

^s^Parental Locus of Control (PLOC)

^t^Treatment As Usual (TAU)

^u^This number represents the sample size of the CA and STD groups only, omitting the TAU sample, which was not of central interest to this review

^v^Note: This number represents the sample size of the CA and STD groups only, omitting the contingency management (CM) and CBT + CM groups, because neither group could serve as an adequate control for the CA group

^w^Outcomes reported are from post-test, as the follow-up data was confounded when participants in either group exchanged reading materials after the post-test measure

^x^Note: This number represents the sample size of the CA and STD groups only, omitting the fact-sheet sample, because this group cannot serve as an adequate control for the CA group

#### Uptake of preventive services

Seven studies attempted to increase uptake of preventive screening services. Jandorf et al. and Holt et al. sought to increase colorectal cancer (CRC) screening in African Americans [51, 52, 66, 67]. No statistically significant improvements were observed in preventive screening uptake. Kalichman et al. sought to increase HIV screening rates and awareness among African American women [46]. Adaptations of their promotional video resulted in an increase in HIV screening and more favorable responses to presenters. Halbert et al. also targeted African American women in an effort to increase genetic screening following a counseling session about breast cancer genes, but found no significant gains for the culturally adapted group [68].

Kreuter et al., Skaer et al., and Wang et al. each attempted to increase mammography rates in African Americans, Latinas with low-incomes, and Chinese Americans, respectively [48, 49, 57, 60–62]. Kreuter et al. found tailoring on both cultural and behavioral variables showed the highest increase in self-reported mammography, but this was not significant relative to behaviorally-tailored materials alone at the 18 month follow-up [60–62]. Wang et al. found that a culturally adapted promotional video was not more effective in increasing mammography compared to control, although mammography uptake was moderated by acculturation status [48, 49]. Skaer et al. observed the largest effect of the included studies: Latina women with low-incomes receiving vouchers for free mammography were over 47 times more likely to receive a mammogram than controls [57].

#### Awareness, knowledge, and attitudes

Six studies aimed to modify recipients’ awareness, knowledge, and/or attitudes as a primary goal. Webb et al. attempted to increase smoking-related disease awareness and perceptions of risk in African Americans who smoke [69]. Culturally adapted materials did not lead to greater knowledge than controls, but did increase risk perceptions and result in stronger intentions to quit. Sanders Thompson, Kalesan, Wells, Williams, and Caito, as well as Holt et al. targeted cancer screening beliefs among African Americans [70, 71]. Sanders Thompson et al. did not observe a statistically significant difference between adapted and control groups [70]. Holt et al. found that recipients in the adapted group reported higher usage of materials, but observed mixed results with regard to self-efficacy [71]. Mohan et al., La Roche et al., and Unger, Cabassa, Molina, Contreras, and Baron each attempted to increase knowledge of medications [59], asthma [38], and depression [72], respectively, and were successful in at least one measure related to knowledge.

#### Smoking behaviors

Four studies attempted to modify smoking behaviors as their primary research goal. Nollen et al., Orleans et al., and Webb et al. targeted materials to African Americans and observed mixed results [73–75]. Nollen et al. found that despite significantly greater usage of adapted materials, there were no statistically significant differences in smoking outcomes [73]. Webb et al. observed greater readiness-to-quit and more quit attempts in the standard group and no difference in abstinence rates between groups [75]. Adapted materials, however, were rated more favorably in several areas. In contrast, Orleans et al. found a significant increase in quitting behaviors among the adapted group, as well as higher rates of smoking abstinence at 12 months [74]. Johnson et al. targeted materials to multicultural schoolchildren for the purpose of smoking prevention [37]. Their adapted education program reduced the odds of smoking by eighth grade when compared to a non-intervention control, whereas the standard anti-smoking curriculum did not. Johnson et al. also observed that their multicultural curriculum was significantly more effective only among Latino students in Latino-dominant schools. Similarly, their standard program was most effective only among Asian-American students within Asian-American/multicultural schools [37].

#### Substance use behaviors

Four studies focused on other substance-use. Burrow-Sanchez and Wrona, and Burrow-Sanchez, Minami, and Hops found no significant group differences in drinking outcomes or treatment satisfaction among Latino adolescents, however, treatment outcome was moderated by recipients’ ethnic identity and measures of *familism* [76, 77]. Lee et al. observed greater reductions in alcohol-induced problem behavior for Latinos in the culturally-adapted motivational-interviewing group [54]. Shoptaw et al. had mixed results with methamphetamine use and HIV-related sexual risk behaviors among gay and bisexual men. The adapted cognitive behavioral therapy (CBT) group achieved higher average Treatment Effectiveness Scores, but also had higher meth-use than the standard group [78].

#### Other health behaviors

Six studies focused on non-substance use health behaviors. Four addressed physical activity in Chinese Americans [79] and African Americans [45, 50, 53]. Activity outcomes did not significantly differ between experimental and control groups in the three studies that assessed activity [45, 50, 79]. Participants in Newton and Perri’s cultural group rated their group leaders as more appreciative than those in the standard group [45]. Three studies targeted fruit and vegetable intake among African Americans: Ard et al. [53], Kreuter et al. [60–62], and Resnicow et al. [63], with Kreuter et al. specifically targeting women. Kreuter et al. reported that cultural and behavioral tailoring of materials resulted in greater increases in recipients’ daily fruit and vegetable intake, but not significantly more so than behavioral tailoring alone [60–62].

#### Mental health

Three studies focused on mental health outcomes. McCabe and colleagues modified Parent Child Interaction Therapy (PCIT) for Mexican American children with externalizing behavioral problems [55, 56], Pan, Huey, and colleagues tailored exposure therapy for Asian Americans with phobias [64, 65], and Hwang et al. targeted depressive symptoms among Asian Americans [80]. Pan et al. found a significantly greater reduction in phobic outcomes in the adapted group at time two compared to the standard exposure treatment, but both groups were comparable at the long term follow-up [64, 65]. Moderator analyses indicate reductions in catastrophic thinking and general fear were greatest for Asian Americans who were less acculturated to American society.

#### Uptake of treatment services

Two studies focused on treatment participation. Gondolf attempted to increase participation in domestic violence counseling. Gondolf found that neither the all-African-American standard counseling nor culturally-focused counseling resulted in increased treatment completion compared to a multicultural, standard-curriculum counseling group. However, for men with high racial identification, the completion rate was between 63% and 65% when data from both adapted conditions were pooled, compared to a 40% completion for men with high racial identification in the multicultural condition [47]. Havranek et al. also targeted African Americans in an attempt to boost self-efficacy and reduce stereotype threat via a values-affirmation exercise. They found that patients receiving the exercise requested and provided more information about their medical condition, and that patient-provider communication was characterized as significantly more positive [58].

### Excluded studies

There were numerous adaptations observed in the literature which were not tested under the stringent design requirements set forth by this review’s inclusion and exclusion criteria. A number of adaptations present in the literature were difficult to isolate in a research design with direct, equivalent group comparison. The stringent criteria also proscribed inclusion of study designs using retrospective or moderator analyses as their sole method of evaluation, as these did not meet the criteria of an intervention being implemented with the clear intention of targeting specific cultural groups. Studies identified as having non-cultural confounds that could be thought to plausibly affect the health outcomes above and beyond the impacts of cultural adaptation limit the ability to effectively analyze the internal validity of cultural adaptations, and are therefore not included within the descriptive portion of this review. However, implications of findings discussed below were compiled upon review of the body of literature encountered throughout the process of this review as a whole.

## Discussion

The included studies differed in number of adaptations, type of adaptations, and the extent of modification, but all sought to improve the experiences and health outcomes of underserved populations through modification of health and mental health services. This review is unique in that it goes beyond a synthesis of culturally tailored interventions and seeks to identify and analyze only studies in which the research design and data analysis support some confidence regarding the validity of the study conclusions. By limiting the studies to those with direct comparisons between culturally adapted interventions and the same interventions in their un-adapted form, the adaptation is truly tested for effectiveness. By limiting the outcomes to those which are experienced by the service recipient, one is not left to guess whether increased sensitivity of the provider actually results in improved experience for the recipient. While other research designs and by extension other reviews may have addressed similar questions, they are constrained by the inability to separate the effects of the adaptation from the effects of the medical or mental health service.

Of course, the very thing that helps isolate the effect of an adaptation requires a highly structured intervention which will not always be reflective of the patients, contexts, and processes found in other settings. The section on limitations details these issues. However the ability to more fully determine the effectiveness of the adaptation and the existence of other reviews of less rigorous approaches (see Additional file 1) weighed heavily in favor of this approach. The breadth of this review also led to the identification of core cultural adaptations that occur across health and mental health services and an examination of their efficacy in various settings.

Casting a wide net resulted in the development of two frameworks with which practitioners, policy makers, and researchers may conceptualize adaptations and outcomes in future work (Tables 3 and 4). The frameworks describe the extent to which cultural modification is possible and will foster more consistent measurement of health experience throughout the identified categories of health and mental health service adaptations and outcomes. Although conceptual frameworks of cultural adaptations are already present within the research literature [18, 41], the framework within Table 3 is distinct in that it goes beyond summaries of stages in the adaptation process and instead offers a concrete list of all conceivable adaptations at different levels of service implementation. Future research can therefore be informed beyond *how to adapt* to *what can be adapted*, and which adaptations can thereafter be evaluated in isolation. See Castro and colleagues’ review of issues and challenges in the design of culturally adapted interventions for more information and guidance [41]. Lastly, Additional file 6 depicts one way in which the adaptation framework can be applied, illustrating the “level of engagement” of recipients with the cultural adaptation.

### Included studies

As has been previously indicated, there appears to be no universally accepted standard for creating or testing cultural adaptations. The majority of studies tested packages of adaptations that included multiple components. Though some studies implemented adaptations in a specific category (e.g., adapting the content), only five tested singular adaptations (e.g., adding graphics into the content). As a result, researchers in such studies could not assign resulting effects to any specific adaptation, but rather evaluated the package of adaptations as a whole.

Of the 31 retained studies, 9 were identified by the research team as having one or more foreseeable, practical impacts on the health experience of their service recipients. Two of these interventions were categorized as *provision of supplemental resources, services, or support*, and each effectively addressed a separate barrier to service uptake. Skaer et al. noted that previous research had indicated that financial concerns were identified as the greatest barrier to mammography screening uptake for Latinas [57]. Their approach was to provide vouchers for free mammography screening to low-income Latina women, which was effective in achieving significantly greater preventive screening. Havranek et al. similarly address barriers to treatment by targeting communication as a barrier to effective treatment uptake for African Americans [58]. Clients received a values-affirmation exercise prior to meeting with their general practitioner that guided them to identify their own values and strengths in an effort to reduce perceived stereotype threat and improve the quality of provider-client interactions. Those who participated in the exercise were more likely to request information regarding their medical condition which in turn enhanced the provider-patient interaction.

Five other studies implemented effective packages of interventions. Orleans et al. implemented a package intervention consisting of both culturally relevant materials and culturally sensitive counselling, which was effective at increasing participants’ smoking quit-rate at 12 months [74]. Lee et al. adapted a motivational interviewing session that resulted in a significant decrease in scores on a scale that measures serious legal and physical harms related to alcohol use (DRInC Impulse scale). The authors also found a difference in the reduction of number of heavy drinking days per month that approached significance (*p* = .082 = .10, f = .33) [54]. A study by Kalichman et al. found that cultural adaptations to their AIDS video resulted in more participants requesting condoms and talking about AIDS with their friends [46]. In addition, only the participants in the group with both adapted content and ethnically matched providers went for HIV testing in the 2 weeks after the intervention. The Multifamily Asthma Group Treatment (MFAGT) implemented by LaRoche et al. was likewise effective; in their study, MFAGT was significantly better at increasing parental asthma knowledge and reducing visits to the emergency department [38]. Lastly, Hwang et al. observed a significant interaction of treatment by time with regard to decreased depression through the use of their culturally adapted CBT program [80].

Two other studies of note did not find significant results regarding health indicators or health behavior outcomes, but did observe differences that could be seen as meaningful to the health experience of service recipients. For instance, although Unger et al. found no statistical differences in willingness to seek help between groups, participants who read the culturally adapted fotonovela reported significantly less stigma regarding antidepressants and mental health care, in addition to increased depression knowledge [72]. Similarly, McCabe et al. found that their culturally adapted Parent Child Interaction Therapy outperformed the standard treatment on all outcomes, with between-groups Cohen *d*’s ranging from .09 to .36, though no differences reached statistical significance [56] (Table 5).

### Excluded studies

The exclusion of studies that used single-group, wait-list, or other non-equivalent designs resulted in the exclusion of some popular and interesting methods of cultural modification from this review. For example, many studies tested the introduction of patient navigators in the service uptake process, but none controlled for both attentional and informational confounding factors inherent in patient navigation. The same was true of educational interventions aimed at improving providers’ cultural competence. The inclusion criteria of this review required that a provider intervention: (a) isolate the cultural adaptation from other interventions and (b) measure providers’ behavioral outcomes or the recipients’ outcomes. None of the identified studies met both of these criteria, as many studies did not utilize adequate controls or assessed only changes in providers’ knowledge, attitudes, and awareness.

Another common cause of omission from this review was a lack of cultural justification for the adaptation, that is, if there was no reason given to indicate that the chosen adaptation would benefit the target population any more than it would benefit the general population. For example, standard psychotherapies were omitted if they were not indicated to be especially efficacious in, or adapted for, a given population. Only studies that supplied cultural justification for supplemental services, such as Skaer et al., were retained. Skaer and colleagues justified their financial intervention with survey research previously undertaken in the community of interest regarding barriers to mammography screening [57]. This process conformed with the intent of this review to identify adaptations that were truly cultural in nature and that were implemented to meet the needs of specific cultural groups.

### Adaptations and efficacy for different health problems

The main method of addressing the health concerns targeted within the retained studies appeared to be reformatting intervention content to contain culture-specific information and themes or convey this content in a manner congruent with the target culture. Despite wide variation in health targets, this basic principle appeared to remain. It was only once studies were classified into preventive- versus treatment-based interventions that any pattern could be discerned. For example, if the goal was cancer prevention, the focus often rested on increasing the knowledge of target populations and a consequent change in behavior such as obtaining screening services.

Two studies specifically targeted medical treatment: Havranek implemented changes to affect the medical treatment dialogue [58] and Mohan implemented changes to medical instructions [59]. No retained studies assessed preventive strategies targeting mental health outcomes, other than Unger et al.’s use of a fotonovela to increase willingness to seek help for depression [72].

### Adaptations and efficacy for different populations

There was no one clear method of adaptation for any specific population or culture but there were a few observed similarities in the values targeted for distinct groups. For example, familism was frequently presented as a value central to Latino culture. At the same time, other studies identified collectivist beliefs as present in many Asian cultures. These orientations may represent similar characteristics with respect to familial relationships and may result in similar adaptations. Observations of this type highlight the fact that race or common ancestry alone is not useful for selecting types of cultural adaptations. Variations within cultures and the degree to which people practice or support values commonly associated with their culture are important in designing effective adaptations. To address this, some researchers have focused specifically on degree of acculturation, while others have attempted to devise more individualized adaptations.

In addition to specific cultural orientations, some providers have also attempted to be more aware of, and responsive to, past traumatization of whole populations such as African Americans in the U.S. [13] and indigenous peoples in Canada and the U.S. [10]. In some cases, the most appropriate goal may be to build trust from the moment of the service user’s introduction to service providers or their organizations, including for example, developing more welcoming physical surroundings. While these changes are not uncommon, no studies that were found isolated and tested such changes.

#### Moderator effects

Occasionally, researchers observed null findings overall, but reported significant interaction effects related to culture. A number of studies found recipients’ level of acculturation to the predominant society moderated the effects of cultural adaptations. For example, Wang et al. found that a culturally adapted video was more effective for women who were lower in acculturation [48]. Pan et al., Gondolf, and Burrow-Sanchez et al. likewise found that adapted programs were more effective amongst Asian Americans less-acculturated to U.S. culture, African Americans with high racial identification, and Latino adolescents with high ethnic identity commitment, respectively [47, 65, 77]. These findings provide some support for tailoring based on level of acculturation, which was featured in three reviewed studies: Huey and Pan and Pan et al., Kreuter et al., and Resnicow et al. [60–65]. Two studies that went on to use cultural tailoring based on acculturation did not find it significantly more effective than non-tailored treatments. Resnicow et al. found that culturally tailored materials based on *ethnic identity* were significantly more effective than standard materials for their Afro-centric subgroup [63].

### A complex issue

The observed interaction effects suggest that treatment efficacy is contingent on multiple variables. One method of specified tailoring observed in the literature is to individualize content based on numerous demographic variables, which could range from ethnicity and age group to preferred communication style and interest in materials [81]. While this review did not focus on individualization, the studies found indicate it may show promise in improving recipient outcomes, and may function best when applied to multiple factors.

Successful adaptation is further complicated by the possibility that research participants may be fundamentally different than those who choose not to participate at all, or those who are never even approached for research. Both groups could be different from research participants on a variety of social, economic, and personal factors. Medical mistrust is one plausible example of the potential unrepresentativeness of research samples; if a person does not trust those in the medical field enough to seek help for their ailments it is unlikely that they would permit medical researchers to study them. Those most in need of culturally competent services may not be involved in the development of culturally competent interventions designed to reach them.

#### Statistical significance

It is possible that some retained studies failed to observe significant effects because the sample population was not as underserved as the initial population of interest. For example, Jandorf and colleagues, in a comparison of completers to drop-outs, noted that all of their participants (experimental and control) had insurance coverage [51]. The implication is that differential access to medical information may not be as pronounced in this sample as it would be in a sample including uninsured individuals.

Another commonly cited explanation for observed results was that the statistical significance of the effects may have been obscured by the nature of the control conditions; rarely is a standard intervention completely culturally insensitive. The retained studies all tested cultural adaptations in isolation, but they often did so above and beyond pre-existing adaptations to improve effectiveness. For instance, Holt et al. conducted both their experimental and control conditions within a church, so even the standard condition was perceived as highly spiritual by recipients [66, 67].

Included studies also tended to focus on statistical significance rather than effect sizes. Small sample sizes in many studies may have made it difficult for small effects to achieve significance—effects which may have practical importance. For example, Lee et al. measured previous-month *heavy drinking days*, and although the outcome was statistically non-significant, there was medium effect size in favor of the adapted group [54]. It should be noted that even minimally increased efficacy could have practical and financial effects when one takes into account the millions of individuals that are currently involved with some form of health services.

### Mechanisms of influence

Although mechanisms of influence are not often elaborated in the research reviewed, it was possible to discern four main pathways by which researchers sought to achieve more effective services for underserved populations: (1) addressing systemic barriers such as location of service, transportation, language, child care, and affordability of services, (2) increasing community engagement to help identify and ameliorate barriers, (3) integrating cultural perspectives and values directly into the intervention, and (4) enhancing the service experience, thereby increasing satisfaction and ideally the likelihood of access, uptake, and follow-through to result in improved health outcomes. These mechanisms are not mutually exclusive but they highlight major pathways to potentially successful intervention (increased access, uptake, follow-through, knowledge, and ultimately, changes in health status or behavior). The included research clearly indicates that one or a combination of the ideal outcomes is not necessarily sufficient to ultimately achieve changes in health. The mechanisms change to some degree with the nature of the desired outcome.

#### Overcoming systemic barriers

Finances, geography, or comprehension ability are some examples of systemic barriers to accessing adequate health care. Addressing these systemic barriers was an important focus in a few of the retained studies. The efficacy of Skaer and colleagues’ mammography vouchers highlights the importance of financial barriers, identified in prior community research as the greatest barrier to treatment for their population of interest [57]. The addition of illustrations to simplify content, and therefore address barriers of communication and understanding, has likewise been shown to be effective in improving some health outcomes, particularly knowledge and understanding [59, 72]. The question then becomes how to translate increased understanding into behavior change, e.g., seeking treatment or adhering to medication [59, 72].

#### Community engagement

Community engagement was often the foundation upon which adapted interventions were developed. Researchers strove to involve cultural consultants and community members in the development of their interventions and focus-group testing of the interventions to ensure their validity [37, 46, 48, 49, 54–56, 60–67, 71, 72, 76, 77, 80]. It was not possible to determine to what degree this engagement involved true partnerships with the community versus less involved approaches such as brief consultation. Such authentic partnerships are often identified as the first step to making services more relevant to diverse peoples [82]. Interestingly, at least one researcher observed the adaptations developed in concert with the community were not noticed by the target population. Pan et al. found in a manipulation check that every participant in the adapted condition believed they were receiving the standard, un-adapted treatment, in spite of the extensive evidence-based cultural adaptations made to psychotherapy [65]. Whether or not recognition of adaptation is associated with success is unknown. In practice, of the studies that included involvement of the community in the development of an intervention, a little less than half were found to be effective in some way [46, 62–65, 71, 72, 76, 80].

#### Integrating cultural context and perspectives with treatment

Some studies attempted to achieve better health outcomes by integrating aspects of culture or cultural understanding with the treatment activity. Four of the nine adapted interventions that had a practical impact on the health outcomes of recipients implemented supplemental cultural components that were not available in the standard intervention that addressed direct needs of the service recipients [54–58]. Seven of the nine implemented packages of adaptations both modified the content and the manner in which the content was delivered. Wording was simplified, group composition was altered, and the treatment procedure changed, among other adaptations [38, 46, 55, 56, 72, 74, 80].

Some attempted to increase the level of interactivity and rapport between provider and recipient, extending the concept of partnership to provider-recipient interactions. Pan et al., for instance, adapted their style of communication (being more directive) to be more congruent with their clients’ expectations [65]. These efforts were not necessarily more effective than standard treatment, for example, Jandorf et al.’s use of peers as navigators [51, 52], but it did result in some distinct findings. For example, although all four studies that used Pathways to Freedom in their interventions [69, 73–75] were judged as statistically effective, it was the increase in quit rate among recipients in the adapted group in Orleans et al. that was considered practically important. Orleans et al. implemented a counseling component in addition to the Pathways to Freedom guide that was culturally adapted to be more interactive [74]. Likewise, the intervention group in the Kalichman et al. study that received both adapted content and a change in manner in service delivery was the only condition that resulted in recipients screening for HIV [46].

The Havranek et al. study sought to improve the quality of provider-recipient interaction. It was the only retained study to examine both the service provider and service recipient outcomes simultaneously, as a pair [58]. A large effect was observed, with increased client communication that resulted in less provider-dominance. This study may suggest that provider competency can be influenced through client-based interventions, but also that provider-client communication can be improved in a dyadic and recursive way. Interventions targeting both service providers and service recipients may not only be able to complement each other, but may also produce interaction effects that have a wider impact than previously expected. Interestingly, the enhanced communication and patients’ increased knowledge about their conditions was not accompanied by requests for more information about services or increases in reported satisfaction or increased trust in the provider.

#### Service satisfaction

Client or patient engagement may be fostered by feelings of satisfaction with the services provided (e.g., [83]). The literature on service satisfaction suggests that the construct is multi-faceted and includes expectations of the service user, the quality of the services received, feelings about the experience, the degree to which the service recipients’ beliefs about the services are confirmed in a positive or negative way, and whether the treatment was fair or equitable [84]. It may be measured globally with respect to overall satisfaction or in more detailed fashion, with indicators of several facets of satisfaction. Although some retained studies assessed service satisfaction, not all studies considered mechanisms of influence related to satisfaction. Burrow-Sanchez et al., for example, measured satisfaction as a health outcome in and of itself as a component of intervention feasibility [76]. In some cases attempts at increasing satisfaction may be ineffective. In the case of Jandorf et al., the professional navigators scored higher in satisfaction, and also resulted in a higher colonoscopy completion rate, though not statistically significant [51]. In studies of cultural adaptations, it is possible that recipients’ expectations may contribute to the improvement of such outcomes as client satisfaction or trust in providers [85] — effects which may go unnoticed in studies that use participant-blinding to enhance internal validity.

### Implications for health policy and service providers

Improved health outcomes for diverse populations can be fostered in many ways. In addition to larger societal changes, such as more equitable access to nutrition, health, and education, providers and policy makers can also influence the service experience and outcomes.

#### The paradox

One of the essential lessons is that wholesale changes in materials provided or even service delivery personnel will not automatically be helpful. The paradox of trying to make services more culturally sensitive is that it can result in over-generalization regarding what is important to service users. The research suggests service recipients in this modern, socially interconnected world likely belong to multiple cultural or social groups simultaneously [86]. No one adaptation or package was found to be a cure-all in this review, nor is it plausible that a single adaptation will ever be the answer to cultural competency. Each theory, each intervention, and each outcome is simply a piece of a larger puzzle that needs to be incrementally assembled to build culturally competent services. It is critically important to be aware of variations within cultural groups. With such awareness, for example, one might identify the locus of the problem in a particular service or geographic area, identify barriers to access and uptake and the existing service elements that reinforce these barriers, and understand the variety of paths to achieving service effectiveness for all concerned.

#### Potential pathways to change

There appear to be numerous possible pathways to success but few, if any, have been systematically replicated over time. For policy makers, one possible first step is to assure that pilot projects have sufficiently controlled studies to inform the question of efficacy. Additionally, reaching out to the community to determine their needs/barriers to service and then clearly addressing those needs begins to build the reciprocal relationship that is necessary at both the community and individual levels of service. Supplemental services to address barriers may provide some hope for change if they are actually responsive to the felt need. Attending to the recipient’s experience with services delivered, whether or not they recognize any cultural adaptations, may also support service engagement and later follow-through. Attention to and reporting of cost analyses in all efforts would be enormously helpful for future decision making.

In terms of practice, service for diverse populations can be optimized by taking great care in individualizing, understanding, and showing respect for each person’s individual needs and barriers [87]. Inquiring about a person’s culture and what is important to them is a major first step. At least one study showed that by strengthening the individual service recipient’s self-assurance and affirming their values, improved communication is possible.

### Interventions not included in the review

The studies reviewed were necessarily limited. Interventions for which the adaptation cannot be isolated or cultural alternatives to standard adaptations that do not have a reasonable comparison (e.g. cultural treatments like First Nations art or elements of tai chi) should not be ignored, and should be interpreted and incorporated into all dialogue relating to cultural competence, albeit in a way that differs from those retained in this review of isolated cultural adaptations. See Additional file 1 for further reviews.

### Implications for health research

The lack of available detail on the adaptations studied was disconcerting during this review. Promoting the use of an adapted intervention requires the provision of sufficient detail such that adaptations can be effectively incorporated into practice, for instance, provision of easy access to treatment manuals to ensure fidelity. Additionally, the implementation of tested interventions within existing systems should be plausible, or at least provide suggestions regarding how different systems could be adapted to incorporate these interventions.

#### Specificity of adaptations and outcomes

Understanding in this field can be improved with specific focus on a variety of health outcomes, using methods that isolate and analyze adaptations differing in number, type, and depth. Such focus will help develop a deeper understanding of recipients’ and providers’ health experiences and maximize the effectiveness of health interventions. Studies that provide more explicit detail and documentation of mechanisms of change (e.g., including client satisfaction) would make major contributions. As in most systematic reviews, the dearth of attention to cost analyses is a hindrance to future progress. Research should be undertaken in a manner that permits and emphasizes the effects that services have on the health experience of their clients, in as much detail as possible throughout their involvement with health services.

#### Building the evidence

Careful, generative research will aid in the systematic development of effective interventions. The lack of research that sufficiently controls for other influences is delaying the development of the most effective cultural adaptations. Further, additional systematic replications with variations in sample and methods may illuminate any potential differential patterns of results related to culture and/or geographical region, help support the development of adaptations that are not amenable to highly controlled conditions, and inspire novel methods of cultural adaptation. Some efforts may actually have a deleterious effect on service recipients, such as inducing stereotype threat rather than combatting it or correcting for it, and have mixed or reverse impacts. Interventions must be shown to be congruent with the needs of the population in which they are being implemented through evidenced based research and thorough needs assessments.

### Limitations of this review

RCTs and very rigorous quasi-experimental designs, by their very nature, limit the participants and contexts within which the studies are conducted. For example, the types of organizations that are able to conduct such a study, the types of adaptations that can be evaluated with tightly controlled research designs, and even the countries that have sufficient resources to run such studies will bias, to some extent, the knowledge resulting from such studies (see, e.g., [88, 89]). This project focused on more stringent designs due to the amount of literature available regarding less rigorous approaches (Additional file 1) and the dearth of information available about actual effectiveness of cultural adaptations. The inclusion of more rigorous studies yielded more information about potential effectiveness of cultural adaptation than is available in other publications to date and complements existing knowledge in the field.

Other limitations stem from the procedures required for such a sizeable study and the limited source of the materials that were qualified for inclusion. For instance, several teams of reviewers conducted the screening. Though several safeguards for consistency were in place, differences among the teams are possible. Tests for publication bias were not conducted due to the extraordinary variety of adaptations, outcomes, and designs used in the studies. Limiting the search to reports in English also precludes all studies published in only non-English languages. Lastly, all 31 retained studies were conducted in the United States, so the application of their results, and the corresponding conclusions of this review, may not generalize to other nations with differing social, political, cultural, and/or economic structures.

## Conclusion

This review identifies the most rigorous research in the field of cultural adaptations of health and mental health services, and presents study findings within two conceptual frameworks. These frameworks allow for more systematic categorization of health outcomes and cultural adaptations to inform and support future research and practice in this area. The results suggest several important directions for development of future practice, policy, and research. For practitioners, the literature suggests that high quality services are the result of engaging communities, understanding the needs and desires of the patient or client populations, and adapting to their needs as much as possible in each service encounter. Policy should likewise be congruent with the needs of those involved, and should be informed by both the impacts on individuals and a macroscopic understanding of local communities as a whole. Policies should maximize benefit and minimize harm for all those they affect.

As efforts to produce culturally competent services continue, future research should focus on the isolated study of cultural adaptations, alone and in packages, to identify which among them augment efficacy. The moderating role of acculturation could be explored to a greater extent to yield a more complete understanding of the role of tailoring in health and mental health. The variations within groups also appears to support the individualization of services. The exploration of which interventions are effective, for whom, and what sort of outcomes they influence continues to be of importance in health and mental health service delivery, and is critical to establishing cultural competence and promoting health and mental health in our diverse, multicultural societies.

## Additional files

Additional file 1: Similar Reviews and Meta-analyses. Other reviews on cultural health disparities and service adaptations located during search and screening process. (DOCX 36 kb)
Additional file 2: Glossary. A list of main terms used in this report with their corresponding operational definition. (DOCX 23 kb)
Additional file 3: Complete Electronic Database Search Strategy for the Systematic Review. A record of search strategy and dates for the electronic database searches. (DOCX 48 kb)
Additional file 4: Grey Literature Sources. A list of sources used at the time of the grey literature search, with year searched if the link changed between 2012 and 2015. (DOCX 20 kb)
Additional file 5: Detailed Table of Tested Adaptations and Outcomes by Report. A table sorting retained studies, their adaptations, and their outcome variables in accordance with the conceptual adaptation and outcome frameworks (Tables 3 and 4). (DOCX 83 kb)
Additional file 6: One Example of a Conceptual Hierarchy of Client Engagement. An example of another potential dimension by which adaptations could be evaluated. (DOCX 20 kb)
Additional file 7: Forest Plot - Intervention Effects by Number of Adapted Elements. A forest plot illustrating treatment efficacy sorted by number of adapted elements. (DOCX 84 kb)

## Abbreviations

ADHD: Attention deficit hyperactivity disorder
CA: Culturally adapted
CBCL: Child behavior checklist
CD: Conduct disorder
CRC: Colorectal cancer
DrInC: Drinkers’ inventory of consequences
DSM-IV TR: Diagnostic and statistical manual of mental disorders, 4th edition text revision
ECBI: Eyberg child behavior inventory
ECI: Early Childhood inventory
FOBT: Fecal occult blood test
ODD: Oppositional defiant disorder
PCIT: Parent child interaction therapy
PI: Principal investigator
PLOC: Parental locus of control
PSI: Parenting stress index
RCT: Randomized controlled trial
STD: Standard
TAU: Treatment as usual
USDHHS: U.S. Department of Health and Human Services

## Footnotes

1 Kalichman et al. tested both an adaptation in isolation, but also a package of adaptations in a second comparison [46]. As a result, this study has been counted twice.
