# Supplementary material for: Cultural adaptations to augment health and mental health services: a systematic review
Source: BMC Health Serv Res. 2017 Jan 5;17:8. doi: 10.1186/s12913-016-1953-x (PMC5217593; doi:10.1186/s12913-016-1953-x)
Supplement: Additional file 1: — Similar Reviews and Meta-analyses. Other reviews on cultural health disparities and service adaptations located during search and screening process. (DOCX 36 kb) [file 12913_2016_1953_MOESM1_ESM.docx]

Additional file 1

Similar Reviews and Meta-analyses

The following reviews of research were located during the search and screened for potentially relevant reports. Although these studies yielded few results that met this study’s stringent criteria, they represent an array of populations, research designs, and adaptations observed in the literature on cultural health disparities and service adaptations.

| Author, Year | Population | Topic | Type of Review |
| --- | --- | --- | --- |
| Allen, 2010 [1] | Service providers | Provider cultural competence | Literature Review |
| Anderson et al., 2003 [2] | Diverse | Organizational cultural competence | Systematic Review |
| Bailey et al., 2005 [3] | Low-income | Preventive interventions (cancer) | Systematic Review |
| Bailey et al., 2009 [4] | Diverse | Culturally adapted asthma interventions | Systematic Review |
| Beach et al., 2005 [5] | Service providers | Provider cultural competence | Systematic Review |
| Beach et al., 2006 [6] | Service providers | Provider cultural competence | Systematic Review |
| Bender et al., 2014 [7] | Asian Americans | Lifestyle interventions | Systematic Review |
| Benish et al., 2011 [8] | Diverse | Culturally adapted psychotherapies | Meta-Analysis |
| Brown, 2007 [9] | Service providers | Culturally appropriate diabetes care | Literature Review |
| Brownstein et al., 2007 [10] | Diverse | Community health worker interventions | Systematic Review |
| Carson et al., 2012 [11] | Aboriginal youths | Culturally appropriate preventive interventions (smoking) | Systematic Review |
| Chipps et al., 2008 [12] | Service providers | Provider cultural competence | Systematic Review |
| Chowdhary et al., 2014 [13] | Diverse | Culturally adapted psychotherapies (depressive disorders) | Systematic Review |
| Corcoran et al., 2012 [14] | Latinas | Preventive interventions (cancer) | Systematic Review |
| Forsetlund et al., 2011 [15] | Diverse | Culturally appropriate health care services | Systematic Review |
| Griner & Smith, 2006 [16] | Diverse | Culturally adapted psychotherapies | Meta-Analysis |
| Glazier et al., 2006 [17] | Diverse | Culturally appropriate diabetes interventions | Systematic Review |
| Gonzales et al., 2014 [18] | Diverse youths | Culturally adapted psycho- and behavioral therapies | Literature Review |
| Goris et al., 2013 [19] | Diverse | Community health worker interventions | Systematic Review |
| Han et al., 2009 [20] | Diverse | Preventive interventions (cancer) | Meta-Analysis |
| Harlow et al., 2014 [21] | Aboriginal youths | Culturally appropriate preventive interventions (suicide) | Systematic Review |
| Hasnain et al., 2009 [22] | Individuals with disabilities | Culturally adapted rehabilitation interventions | Systematic Review |
| Hawthorne et al., 2010 [23] | Diverse | Culturally appropriate diabetes education | Systematic Review |
| Henderson, et al., 2011 [24] | Diverse | Culturally appropriate preventive interventions (chronic disease) | Systematic Review |
| Herbst et al., 2007 [25] | Latinos | Preventive interventions (HIV) | Meta-Analysis |
| Jackson, 2009 [26] | Diverse youths | Culturally appropriate psychosocial interventions | Systematic Review |
| Legler et al., 2002 [27] | Diverse | Preventive interventions (cancer) | Literature Review |
| Lie et al., 2010 [28] | Service providers | Provider cultural competence | Systematic Review |
| Maramba & Hall, 2002 [29] | Diverse | Ethnic matching of providers to clients | Meta-Analysis |
| Maulsby et al., 2013 [30] | Gay/Bisexual African Americans | Preventive interventions (HIV) | Systematic Review |
| Metzger et al., 2013 [31] | African American youths | Culturally appropriate behavioral interventions | Systematic Review |
| Millan-Ferro & Caballero, 2007 [32] | Latinos | Culturally appropriate diabetes interventions | Literature Review |
| Naylor et al., 2012 [33] | Diverse | Culturally appropriate preventive interventions (cancer) | Systematic Review |
| Nierkens et al., 2013 [34] | Diverse | Culturally adapted smoking interventions | Systematic Review |
| Pottie et al., 2013 [35] | Diverse | Culturally appropriate diabetes education | Literature Review |
| Price et al., 2005 [36] | Service providers | Provider cultural competence | Systematic Review |
| Pugh, 2007 [37] | Service providers | Provider cultural competence | Dissertation |
| Shin et al., 2005 [38] | African Americans | Racial matching of providers to clients | Meta-Analysis |
| Smith et al., 2006 [39] | Service providers | Provider cultural competence | Meta-Analysis |
| Wilson & Miller, 2003 [40] | Diverse | Culturally appropriate preventive interventions (HIV) | Literature Review |

References

1. Allen J. Improving cross-cultural care and antiracism in nursing education: A literature review. Nurse Education Today. 2010:30:314-320. doi:10.1016/j.nedt.2009.08.007
2. Anderson LM, Scrimshaw SC, Fullilove MT, Fielding JE, Normand J, the Task Force on Community Preventive Services. Culturally competent healthcare systems: A systematic review. American Journal of Preventive Medicine. 2003;24(3S):68-79; doi:10.1016/S0749-3797
3. Bailey EJ, Cates CJ, Kruske SG, Morris PS, Brown N, Chang AB. Culture-specific programs for children and adults from minority groups who have asthma. Cochrane Database of Systematic Reviews (Online). 2009;(2):CD006580; doi: 10.1002/14651858
4. Bailey TM, Delva J, Gretebeck K, Siefert K, Ismail A. A systematic review of mammography educational interventions for low-income women. American Journal of Health Promotion. 2005;20:96-107. http://www.ncbi.nlm.nih.gov/pmc/articles/PMC1820866/
5. Beach MC, Gary TL, Price EG, Robinson K, Gozu A, Palacio A, et al. Improving health care quality for racial/ethnic minorities: A systematic review of the best evidence regarding provider and organization interventions. BMC Public Health. 2006;6 http://www.ncbi.nlm.nih.gov/pmc/articles/PMC1525173/pdf/1471-2458-6-104.pdf
6. Beach MC, Price EG, Gary TL, Robinson KA, Gozu A, Palacio A, et al. Cultural competency: A systematic review of health care provider educational interventions. Medical Care. 2005;43:356-373.
7. Bender MS, Choi J, Won GY, Fukuoka Y. Randomized controlled trial lifestyle interventions for Asian Americans: A systematic review. Preventive Medicine. 2014;67: 171-181; doi:10.1016/j.ypmed.2014.07.034
8. Benish SG, Quintana S, Wampold BE. Culturally adapted psychotherapy and the legitimacy of myth: A direct-comparison meta-analysis. Journal of Counseling Psychology. 2011;58:279-289; doi:10.1037/a0023626
9. Brown AF. Patient, system and clinician level interventions to address disparities in diabetes care. Current Diabetes Reviews. 2007;3:244-248; http://www.ncbi.nlm.nih.gov/pubmed/18220681
10. Brownstein JN, Chowdhury FM, Norris SL, Horsley T, Jack L Jr., Zhang X, Satterfield D. Effectiveness of community health workers in the care of people with hypertension. Effectiveness of community health workers in the care of people with hypertension. American Journal of Preventive Medicine. 2007;32:435-447; doi:10.1016/j.amepre.2007.01.011
11. Carson KV, Brinn MP, Labiszewski NA, Peters M, Chang AB, Veale A, et al. Interventions for tobacco use prevention in Indigenous youth. Cochrane Database of Systematic Reviews. 2012;15:CD009325; doi:10.1002/14651858.CD009325.pub2.
12. Chipps JA, Simpson B, Brysiewicz P. The effectiveness of cultural-competence training for health professionals in community-based rehabilitation: a systematic review of literature. Worldviews on Evidence Based Nursing. 2008;5:85-94; doi:10.1111/j.1741-6787.2008.00117.x
13. Chowdhary N, Jotheeswaran AT, Nadkarni A, Hollon SD, King M, Jordans JD, et al. The methods and outcomes of cultural adaptations of psychological treatments for depressive disorders: A systematic review. Psychological Medicine. 2014;44:1131–1146.
14. Corcoran J, Dattalo P, Crowley M. Cervical cancer screening interventions for U.S. Latinas: A systematic review. Health & Social Work. 2012;37:197-205; http://www.ncbi.nlm.nih.gov/pubmedhealth/PMH0054059/
15. Forsetlund L, Eike MC, Vist GE. Effect of interventions to improve health care services for minority populations. Norsk Epidemiologi. 2010;20:41-52; http://www.researchgate.net/publication/50282117_Effect_of_interventions_to_improve_health_care_services_for_ethnic_minority_populations
16. Griner D, Smith TB. Culturally adapted mental health intervention: A meta-analytic review. special issue: Culture, race, and ethnicity in psychotherapy. Psychotherapy. 2006;43:531-548; http://psycnet.apa.org/index.cfm?fa=buy.optionToBuy&id=2006-23019-014
17. Glazier RH, Bajcar J, Kennie NR, Willson K. A systematic review of interventions to improve diabetes care in socially disadvantaged populations. Diabetes Care. 2006;29:1675-88.
18. Gonzales NA, Lau AS, Murry VM, Piña AA, Barrera M Jr. Culturally adapted preventive interventions for children and youth. 2014; http://prevention.psu.edu/events/documents/Gonzalesetal.CulturalAdaptationChapter.pdf
19. Goris J, Komaric N, Guandalini A, Francis D, Hawes E. Effectiveness of multicultural health workers in chronic disease prevention and self-management in culturally and linguistically diverse populations: A systematic literature review. Australian Journal of Primary Health. 2013;19:14-37; doi:10.1071/PY11130
20. Han HR, Lee JE, Kim J, Hedlin HK, Song H, Kim MT. A meta-analysis of interventions to promote mammography among ethnic minority women. Nursing Research and Practice. 2009;58:246-254; doi:10.1097/NNR.0b013e3181ac0f7f
21. Harlow A F, Bohanna I, Clough A. A systematic review of evaluated suicide prevention programs targeting indigenous youth. Crisis. 2014;35; doi:10.1027/0227-5910/a000265
22. Hasnain R, Kondratowicz DM, Portillo N, Borokhovski E, Balcazar F, Johnson T, et al. The use of culturally adapted competency interventions to improve rehabilitation service outcomes for culturally diverse individuals with disabilities. Submitted to the Campbell Collaboration, Education Coordinating Group. 2009.
23. Hawthorne K, Robles Y, Cannings-John R, Edwards AGK. Culturally appropriate health education for type 2 diabetes in ethnic minority groups: A systematic and narrative review of randomized controlled trials. Diabetic Medicine : A Journal of the British Diabetic Association. 2010;27:613-23; doi:10.1111/j.1464-5491.2010.02954.x
24. Henderson S, Kendall E, See L. The effectiveness of culturally appropriate interventions to manage or prevent chronic disease in culturally and linguistically diverse communities: A systematic literature review. Health and Social Care in the Community. 2011;19:225-249; doi: 10.1111/j.1365-2524.2010.00972.x
25. Herbst JH, Kay LS, Passin WF, Lyles CM, Crepaz N, Marin, BV. A systematic review and meta-analysis of behavioral interventions to reduce HIV risk behaviors of Hispanics in the united states and Puerto Rico. AIDS and Behavior. 2007;11:25-47; doi:10.1007/s10461-006-9151-1
26. Jackson KF. Building cultural competence: A systematic evaluation of the effectiveness of culturally sensitive interventions with ethnic minority youth. Children and Youth Services Review. 2009;31:1192-1198; doi:10.1016/j.childyouth.2009.08.001
27. Legler J, Meissner HI, Coyne C, Breen N, Chollette V, Rimer BK. The effectiveness of interventions to promote mammography among women with historically lower rates of screening. Cancer Epidemiology, Biomarkers & Prevention. 2002;11:59-71; http://www.ncbi.nlm.nih.gov/pubmed/11815402
28. Lie DA, Lee-Rey E, Gomez A, Bereknyei S, Braddock III CH. Does cultural competency training of health professionals improve patient outcomes? A systematic review and proposed algorithm for future research. Journal of General Internal Medicine. 2010;26:317-325; doi:10.1007/s11606-010-1529-0
29. Maramba GG, Hall GC. Meta-analysis of ethnic match as a predictor of dropout, utilization, and level of functioning. Cultural Diversity & Ethnic Minority Psychology. 2002;8:290-297; doi:10.1037//1099-9809.8.3.290
30. Maulsby C, Millett G, Lindsey K, Kelley R, Johnson K, Montoya D, Holtgrave D. A systematic review of HIV interventions for black men who have sex with men (MSM). BMC Public Health. 2013;13:1-13; doi:10.1186/1471-2458-13-625
31. Metzger I, Cooper SM, Zarrett N, Flory K. Culturally sensitive risk behavior prevention programs for African American adolescents: A systematic analysis. Clinical Child and Family Psychology Review. 2013;16:187-212; doi:10.1007/s10567-013-0133-3
32. Millan-Ferro A, Caballero AE. Cultural approaches to diabetes self-management programs for the Latino community. Issues in the Nutritional Treatment of Diabetes. 2007:391-397; doi:10.1007%2Fs11892-007-0064-9
33. Naylor K, Ward J, Polite BN. Interventions to improve care related to colorectal cancer among racial and ethnic minorities: A systematic review. Journal of General Internal Medicine. 2012;27:1033-1046; doi:10.1007/s11606-012-2044-2
34. Nierkens V, Hartman MA, Nicolaou M, Vissenberg C, Beune EJAJ, Hosper K, et al. Effectiveness of cultural adaptations of interventions aimed at smoking cessation, diet, and/or physical activity in ethnic minorities. A systematic review. PLOS One. 2013;8:e73373; doi:10.1371/journal.pone.0073373
35. Pottie K, Hadi A, Chen J, Welch V, Hawthorne K. Realist review to understand the efficacy of culturally appropriate diabetes education programmes. Diabetic Medicine. 2013;30:1017-1025; doi:10.1111/dme.12188
36. Price EG, Beach MC, Gary TL, Robinson KA, Gozu A, Palacio A, et al. A systematic review of the methodological rigor of studies evaluating cultural competence training of health professionals. Academic Medicine. 2005;80:578-586; http://www.ncbi.nlm.nih.gov/pubmed/15917363
37. Pugh PA. Effective educational delivery methods: A meta-analysis of cultural competence education for health care professionals. (Doctoral dissertation, Capella University). 2007; http://proquest.umi.com/pqdlink?Ver=1&Exp=09-21-2015&FMT=7&DID=1441235791&RQT=309&attempt=1&cfc=1
38. Shin SM, Levy RJ, Chow C, Camacho-Gonsalvez T, Allen IE, Leff HS. A meta-analytic review of racial-ethnic matching for African American and Caucasian American clients and clinicians. Journal of Counseling Psychology. 2005;52:45-56; doi:10.1037/0022-0167.52.1.45
39. Smith T, Constantine M, Dunn T, Dinehart J, Montoya J. Multicultural education in the mental health professions: A meta-analytic review. Journal of Counseling Psychology. 2006;53:132-145; doi:10.1037/0022-0167.53.1.132
40. Wilson BD, Miller RL. Examining strategies for culturally grounded HIV prevention: A review. AIDS Education and Prevention. 2003;15:184-202; http://www.ncbi.nlm.nih.gov/pubmed/12739794
