# Supplementary material for: Cultural adaptations to augment health and mental health services: a systematic review
Source: BMC Health Serv Res. 2017 Jan 5;17:8. doi: 10.1186/s12913-016-1953-x (PMC5217593; doi:10.1186/s12913-016-1953-x)
Supplement: Additional file 2: — Glossary. A list of main terms used in this report with their corresponding operational definition. (DOCX 23 kb) [file 12913_2016_1953_MOESM2_ESM.docx]

Additional file 2

Glossary

| Term | Definition |
| --- | --- |
| Cultural adaptation | Changes made to a service to improve the experience or outcomes of diverse racial, ethnic, and cultural groups, which incorporate aspects of their culture, or is justified by research conducted among members of that cultural community. |
| Cultural appropriate-ness | Represents the “delivery of programmes and services so that they are consistent with the communication styles, meaning systems and social networks of clients, or programme participants, and other stakeholders” [1, p. 1]. |
| Cultural competence | Knowledge of others’ cultures, focusing on an attitude of openness and humility, honoring and respecting others’ beliefs, and development of “the capacity to function effectively as an individual and organization within the context of the cultural beliefs, behaviors, and needs presented by consumers and their communities” [2]. |
| Cultural safety | Focus on structural barriers to care, such as governing policies and the context of the institution enabling people of all groups to feel safe in that environment [3]. |
| Culture | Patterns of “ideas and practices” that are “attached to all the important social distinctions in our lives – race and ethnicity, but also social class, gender, religion, birth cohort, region of the country, and so on”, conveying “what is and is not good, valuable, and worth doing” [4, p. 78]. |
| Ethnicity | A “dynamic set of historically derived and institutionalized ideas and practices that (1) allows people to identify or be identified with groupings of people on the basis of presumed, and usually claimed, commonalities, including several of the following: language, history, nation or region of origin, customs, ways of being, religion, names, physical appearance and/or ancestry group; (2) when claimed, confers a sense of belonging, pride, and motivation; (3) can be a source of collective and individual identity” [4, p. 22]. |
| Race | A “dynamic set of historically derived and institutionalized ideas and practices that: (1) sorts people into ethnic groups according to perceived physical and behavioral human characteristics that are often imagined to be negative, innate, and shared (2) associates differential value, power, and privilege with these characteristics; establishes a hierarchy among the different groups; and confers opportunity accordingly” [4, p. 21]. |

References

1. Thomas DR. Evaluating the cultural appropriateness of service delivery in multi-ethnic communities. Paper presented at the 2002 Australasian Evaluation Society International Conference in Wollongong Australia. 2002; <http://www.evaluationcanada.ca/distribution/20021030_thomas_david.pdf>
2. U. S. Department of Health and Human Services, Office of Minority Health. National standards for culturally and linguistically appropriate services in health care: Final report. Washington, DC: Author. 2001; http://minorityhealth.hhs.gov/assets/pdf/checked/finalreport.pdf
3. Polaschek NR. Cultural safety: A new concept in nursing people of different ethnicities. Journal of Advanced Nursing. 2008;27:452–457; doi:10.1046/j.1365-2648.1998.00547.x
4. Markus HR, Moya PML. (Eds.). Doing race: 21 essays for the 21st century. New York: W W Norton & Company. 2010.
