# Supplementary material for: Cultural adaptations to augment health and mental health services: a systematic review
Source: BMC Health Serv Res. 2017 Jan 5;17:8. doi: 10.1186/s12913-016-1953-x (PMC5217593; doi:10.1186/s12913-016-1953-x)
Supplement: Additional file 3: — Complete Electronic Database Search Strategy for the Systematic Review. A record of search strategy and dates for the electronic database searches. (DOCX 48 kb) [file 12913_2016_1953_MOESM3_ESM.docx]

Additional file 3

Complete Electronic Database Search Strategy for the Systematic Review

**Electronic Search Dates**

MEDLINE on OVID: 1946—August 27, 2011; August 28, 2011—June 22, 2012; June 23, 2012—January 4, 2015.

PsycINFO on EBSCO: 1887—August 29, 2011; August 30, 2011—June 22, 2012; June 23, 2012—January 4, 2015.

CINAHL on EBSCO: 1982—August 30, 2011; September 1, 2011—June 22, 2012; June 23, 2012—January 4, 2015

EMBASE on OVID: 1974—September 7, 2011; September 8, 2011—June 22, 2012; June 23, 2012—January 4, 2015

**Search Strategy**

**Cultural** **Study Design**

**RCTs and quasi-experimental designs**

**Cultural competence, appropriateness, safety, etc.**

**Limits**

Dates: 1950 – current

Language: English

Abstracts must be available

Studies: evaluations, RCTs, observational studies, qualitative studies

**Formats**

Journal articles, books, theses, dissertations, grey literature (policy papers)

**Databases**

MEDLINE, EMBASE, CINHAL, PsycINFO

# Search Terms

## Cultural Competence, Appropriateness, or Safety

Cultural terms may include a combination of thesaurus and free-text terms. Team members were asked to suggest appropriate terms that represent this concept from their respective disciplines as terminology differs to some extent between disciplines.

**Example of terms reviewed for inclusion:**

| Cultural competence | Cultural awareness | Racial bias | Linguistic competence |
| --- | --- | --- | --- |
| Cultural safety | Cultural sensitivity | Racial disparity | Linguistically appropriate |
| Cultural appropriate | Culturally adapted | Racial disparities | Multicultural competence |
| Cultural factors | Cultural responsiveness | Ethnic bias | Ethnic disparities |

## Study Design

The terms for study design and how they are indexed in the literature varies between disciplines. Team members will be asked to suggest terms that represent the different study designs within their field to ensure appropriate terms are captured within the searches.

## Randomized Controlled Trials (RCTs)

Search filters from SIGN (Scottish Intercollegiate Guidelines Network) may be used for RCTs.

**As an example, the RCT filter for MEDLINE is as follows:**

1. Randomized Controlled Trials as Topic/
2. randomized controlled trial/
3. Random Allocation/
4. Double Blind Method/
5. Single Blind Method/
6. clinical trial/
7. clinical trial, phase i.pt
8. clinical trial, phase ii.pt
9. clinical trial, phase iii.pt
10. clinical trial, phase iv.pt
11. controlled clinical trial.pt
12. randomized controlled trial.pt
13. multicenter study.pt
14. clinical trial.pt
15. exp Clinical Trials as topic/
16. or/1-15
17. (clinical adj trial$).tw
18. ((singl$ or doubl$ or treb$ or tripl$) adj (blind$3 or mask$3)).tw
19. PLACEBOS/
20. placebo$.tw
21. randomly allocated.tw
22. (allocated adj2 random$).tw
23. or/17-22
24. 16 or 23
25. case report.tw
26. letter/
27. historical article/
28. or/25-27
29. 24 not 28

**SEARCHES CONDUCTED ARE INCLUDED IN THE FOLLOWING PAGES**

**Database: Ovid MEDLINE(R) 1948 to Present with Daily Update**

**Search Strategy:**

1 Cultural Competency/

2 Cultural Diversity/

3 Cultural Characteristics/

4 Acculturation/

5 Healthcare Disparities/ and (racial or race or ethnic$ or minorit$ or cultur$).tw.

6 (Multicultural adj competen$).tw.

7 (Linguistic$ adj2 (competence or appropriate)).tw.

8 communication barriers/ and (racial or race or ethnic$ or minorit$ or cultur$).tw.

9 ("language barriers" and (racial or race or ethnic$ or minorit$ or cultur$)).tw.

10 (bilingual and (racial or race or ethnic$ or minorit$ or cultur$)).tw.

11 ((trans adj cultural) or transcultural).tw.

12 ((cross adj cultural) or crosscultural).tw.

13 ((inter adj cultural) or intercultural).tw.

14 ((health adj2 ineq$) and (racial or race or ethnic$ or minorit$)).tw.

15 decolonization.tw.

16 Disproportionality.tw.

17 ethnocentrism.tw.

18 bicultural.tw.

19 "Patient Acceptance of Health Care"/eh [Ethnology]

20 ((racial$ or race or ethnic$ or minorit$ or intergroup or "inter group") adj2 (bias$ or disparit$ or sensitiv$)).tw.

21 (cultur$ adj (safety or competen$ or diversit$ or factor? or sensitiv$ or awareness or influence$ or appropriate or responsiveness or adapt$ or knowledge or specific or focus$ or consideration$ or grounded or informed or tailor$ or relevant or congruen$ or consisten$ or ident$ or socializ$)).tw.

22 or/1-21

23 Randomized Controlled Trials as Topic/

24 randomized controlled trial/

25 Random Allocation/

26 Double Blind Method/

27 Single Blind Method/

28 clinical trial/

29 clinical trial, phase i.pt.

30 clinical trial, phase ii.pt.

31 clinical trial, phase iii.pt.

32 clinical trial, phase iv.pt.

33 controlled clinical trial.pt.

34 randomized controlled trial.pt.

35 multicenter study.pt.

36 clinical trial.pt.

37 exp Clinical Trials as topic/

38 or/23-37

39 (clinical adj trial$).tw.

40 ((singl$ or doubl$ or treb$ or tripl$) adj (blind$3 or mask$3)).tw.

41 PLACEBOS/

42 placebo$.tw.

43 randomly allocated.tw.

44 (allocated adj2 random$).tw.

45 or/39-44

46 38 or 45

47 case report.tw.

48 letter/

49 historical article/

50 or/47-49

51 46 not 50

52 (((nonequivalent or non equivalent) adj3 control$) or posttest$ or post test$ or pre test$ or pretest$ or quasi experiment$ or quasiexperiment$ or timeseries or time series).tw.

53 51 or 52

54 22 and 53

55 limit 54 to (abstracts and english language)

***************************

**Database: Embase <1980 to 2011 September 06>**

**Search Strategy:**

1 cultural bias/

2 cultural competence/

3 cultural factor/

4 cultural safety/

5 cultural sensitivity/

6 health care disparity/ and (racial or race or ethnic$ or minorit$ or cultur$).tw.

7 ((communication adj2 barriers) and (racial or race or ethnic$ or minorit$ or cultur$)).tw.

8 ((language adj2 barriers) and (racial or race or ethnic$ or minorit$ or cultur$)).tw.

9 (bilingual and (racial or race or ethnic$ or minorit$ or cultur$)).tw.

10 ((trans adj cultural) or transcultural).tw.

11 ((cross adj cultural) or crosscultural).tw.

12 ((inter adj cultural) or intercultural).tw.

13 ((health adj2 ineq$) and (racial or race or ethnic$ or minorit$)).tw.

14 decolonization.tw.

15 Disproportionality.tw.

16 ethnocentri$.tw.

17 bicultural.tw.

18 ((racial$ or race or ethnic$ or minorit$ or intergroup or "inter group") adj2 (bias$ or disparit$ or sensitiv$)).tw.

19 (cultur$ adj (safety or competen$ or diversit$ or factor? or sensitiv$ or awareness or influence$ or appropriate or responsiveness or adapt$ or knowledge or specific or focus$ or consideration$ or grounded or informed or tailor$ or relevant or congruen$ or consisten$ or ident$ or socializ$)).tw.

20 or/1-19

21 quasi experimental study/

22 ((control or comparison) adj group).tw.

23 (((nonequivalent or non equivalent) adj3 control$) or posttest$ or post test$ or pre test$ or pretest$ or quasi experiment$ or quasiexperiment$ or timeseries or time series).tw.

24 or/21-23

25 Clinical trial/

26 Randomized controlled trial/

27 Randomization/

28 Single blind procedure/

29 Double blind procedure/

30 Crossover procedure/

31 Placebo/

32 Randomi?ed controlled trial$.tw.

33 Rct.tw.

34 (allocat$ adj2 random$).tw.

35 Single blind$.tw.

36 Double blind$.tw.

37 ((treble or triple) adj blind$).tw.

38 Placebo$.tw.

39 Prospective study/

40 or/25-39

41 Case study/

42 Case report.tw.

43 Abstract report/ or letter/

44 or/41-43

45 40 not 44

46 24 or 45

47 20 and 46

48 limit 47 to (abstracts and english language)

***************************

**EBSCO HOST - HEALTH**

| **#** | **Query** | **Limiters/Expanders** | **LastRunVia** |
| --- | --- | --- | --- |
| S11 | S6andS9 | Limiters -Scholarly (Peer | Interface-EBSCOhost |
|  |  | Reviewed)Journals; | SearchScreen-Advanced |
|  |  | AbstractAvailable; | Search |
|  |  | EnglishLanguage; | Database-CINAHLwithFull |
|  |  | ExcludeMEDLINE | Text |
|  |  | records |  |
|  |  | Searchmodes - |  |
|  |  | Boolean/Phrase |  |
| S10 | S6andS9 | Searchmodes - | Interface-EBSCOhost |
|  |  | Boolean/Phrase | SearchScreen-Advanced |
|  |  |  | Search |
|  |  |  | Database-CINAHLwithFull |
|  |  |  | Text |
| S9 | S7orS8 | Searchmodes - | Interface-EBSCOhost |
|  |  | Boolean/Phrase | SearchScreen-Advanced |
|  |  |  | Search |
|  |  |  | Database-CINAHLwithFull |
|  |  |  | Text |
| S8 | (MH"Quasi-Experimental | Searchmodes - | Interface-EBSCOhost |
|  | Studies")OR("non | Boolean/Phrase | SearchScreen-Advanced |
|  | equivalentcontrol*"or |  | Search |
|  | “nonequivalentcontrol*”) |  | Database-CINAHLwithFull |
|  | OR(“controlgroup”or |  | Text |
|  | “comparisongroup”)OR |  |  |
|  | (posttest*or"posttest*" |  |  |
|  | )OR(pretest*or"pre |  |  |
|  | test*")OR( |  |  |
|  | quasiexperiment*or |  |  |
|  | "quasiexperiment*")OR |  |  |
|  | ("timeseries"or |  |  |
|  | timeseries ) |  |  |
| S7 | (MH"ClinicalTrials+")OR | Searchmodes - | Interface-EBSCOhost |
|  | ("randomizedcontrolled | Boolean/Phrase | SearchScreen-Advanced |
|  | trial*"orRCT)OR |  | Search |
|  | “clinicaltrial*”OR |  | Database-CINAHLwithFull |
|  | “multicenter study” OR ( single blind" or "double blind" or "triple blind" ) OR placebo* OR ( “random* allocat*” or “allocate* " |  | Text |
|  | random*” ) |  |  |
| S6 | S1 or S2 or S3 or S4 or | Search modes - | Interface -EBSCOhost |
|  | S5 | Boolean/Phrase | Search Screen -Advanced Search |
|  |  |  | Database -CINAHL with Full |
|  |  |  | Text |
| S5 | ( decoloniz* or disproportionality or ethnocentri* or bicultural* | Search modes -Boolean/Phrase | Interface -EBSCOhost Search Screen -Advanced Search |
|  | ) OR ( “trans cultural” or transcultural ) OR ( “inter cultural” or intercultural ) OR ( “cross cultural” or crosscultural ) |  | Database -CINAHL with Full Text |
| S4 | ( communication barriers" and (racial or race or ethnic* or minorit* " | Search modes -Boolean/Phrase | Interface -EBSCOhost Search Screen -Advanced Search |
|  | or cultur*) ) OR ( language barriers" and (racial or race or ethnic* or minorit* or cultur*) ) OR ( bilingual and (racial or race or ethnic* or minorit* " |  | Database -CINAHL with Full Text |
|  | or cultur*) ) OR linguistic* competen*" OR "linguistic* appropriat*" " |  |  |
| S3 | “cultur* safety” or “cultur* competen*” or “cultur* diversit*” or “cultur* | Search modes -Boolean/Phrase | Interface -EBSCOhost Search Screen -Advanced Search |
|  | factor*” or “cultur* aware*” |  | Database -CINAHL with Full |
|  | or “cultur* influence*” or |  | Text |
|  | “cultur* appropriate” or “cultur* responsive*” or “cultur* adapt*” or “cultur* knowledge*” or “cultur* |  |  |
|  | specific” or “cultur* focus*” or “cultur* |  |  |
|  | considerat*” or “cultur* |  |  |
|  | grounded” or “cultur* informed” or “cultur* |  |  |
|  | tailor*” or “cultur* relevant” |  |  |
|  | or “cultur* congruen*” or “cultur* consisten*” or |  |  |
|  | “cultur* ident*” or “cultur* |  |  |
|  | socializ*” |  |  |
| S2 | (disparit* or ineq* or bias or sensitiv*) and (racial or race or ethnic* or minorit* | Search modes -Boolean/Phrase | Interface -EBSCOhost Search Screen -Advanced Search |
|  | or cultur* or intergroup or “inter group”) |  | Database -CINAHL with Full Text |
| S1 | (MH Cultural Bias") OR (MH "Cultural Safety") OR (MH "Cultural Sensitivity") OR (MH "Cultural Competence") OR (MH "Transcultural Care") OR (MH "Acculturation") " | Search modes -Boolean/Phrase | Interface -EBSCOhost Search Screen -Advanced Search Database -CINAHL with Full Text |

**EBSCO HOST – MENTAL HEALTH**

| **#** | **Query** | **Limiters/Expanders** | **Last Run Via** |
| --- | --- | --- | --- |
| S12 | S7 and S10 | Limiters -Publication | Interface -EBSCOhost |
|  |  | Type: Peer Reviewed Journal; English Search modes -Boolean/Phrase | Search Screen -Advanced Search Database -PsycINFO |
| S11 | S7 and S10 | Search modes - | Interface -EBSCOhost |
|  |  | Boolean/Phrase | Search Screen -Advanced Search |
|  |  |  | Database -PsycINFO |
| S10 | S8 or S9 | Search modes - | Interface -EBSCOhost |
|  |  | Boolean/Phrase | Search Screen -Advanced Search |
|  |  |  | Database -PsycINFO |
| S9 | ( non equivalent control*" or "nonequivalent control*" ) OR ( “control group” or “comparison group” ) OR ( posttest* or "post test*" ) OR ( pretest* or "pre test*" ) OR ( quasiexperiment* or "quasi experiment*" ) OR ( "time series" or timeseries ) " | Search modes -Boolean/Phrase | Interface -EBSCOhost Search Screen -Advanced Search Database -PsycINFO |
| S8 | ( randomized controlled trial*" or RCT ) OR “clinical tiral*” OR " | Search modes -Boolean/Phrase | Interface -EBSCOhost Search Screen -Advanced Search |
|  | “multicenter study” OR ( single blind" or "double blind" or "triple blind" ) OR placebo* OR ( “random* allocat*” or “allocate* " |  | Database -PsycINFO |
|  | random*” ) |  |  |
| S7 | S1orS2orS3orS4or | Searchmodes- | Interface-EBSCOhost |
|  | S5orS6 | Boolean/Phrase | SearchScreen-Advanced |
|  |  |  | Search |
|  |  |  | Database-PsycINFO |
| S6 | (decoloniz*or | Searchmodes- | Interface-EBSCOhost |
|  | disproportionalityor | Boolean/Phrase | SearchScreen-Advanced |
|  | ethnocentri*orbicultural*) |  | Search |
|  | OR(“transcultural”or |  | Database-PsycINFO |
|  | transcultural)OR(“inter |  |  |
|  | cultural”orintercultural) |  |  |
|  | OR(“crosscultural”or |  |  |
|  | crosscultural) |  |  |
| S5 | ("communication | Searchmodes- | Interface-EBSCOhost |
|  | barriers"and(racialor | Boolean/Phrase | SearchScreen-Advanced |
|  | raceorethnic*orminorit* |  | Search |
|  | orcultur*))OR( |  | Database-PsycINFO |
|  | "languagebarriers"and |  |  |
|  | (racialorraceorethnic* |  |  |
|  | orminorit*orcultur*))OR |  |  |
|  | (bilingualand(racialor |  |  |
|  | raceorethnic*orminorit* |  |  |
|  | orcultur*))OR"linguistic* |  |  |
|  | competen*"OR |  |  |
|  | "linguistic*appropriat*" |  |  |
| S4 | “cultur*safety”or“cultur* | Searchmodes- | Interface-EBSCOhost |
|  | competen*”or“cultur* | Boolean/Phrase | SearchScreen-Advanced |
|  | diversit*”or“cultur* |  | Search |
|  | factor*”or“cultur*aware*” |  | Database-PsycINFO |
|  | or“cultur*influence*”or |  |  |
|  | “cultur*appropriate”or |  |  |
|  | “cultur*responsive*”or |  |  |
|  | “cultur*adapt*”or“cultur* |  |  |
|  | knowledge*”or“cultur* |  |  |
|  | specific”or“cultur* |  |  |
|  | focus*”or“cultur* |  |  |
|  | considerat*”or“cultur* |  |  |
|  | grounded”or“cultur* |  |  |
|  | informed”or“cultur* |  |  |
|  | tailor*”or“cultur*relevant” |  |  |
|  | or“cultur*congruen*”or |  |  |
|  | “cultur*consisten*”or |  |  |
|  | “cultur*ident*”or“cultur* |  |  |
|  | socializ*” |  |  |
| S3 | (disparit*orineq*orbias | Searchmodes- | Interface-EBSCOhost |
|  | orsensitiv*)and(racialor | Boolean/Phrase | SearchScreen-Advanced |
|  | raceorethnic*orminorit* |  | Search |
|  | orcultur*orintergroupor |  | Database-PsycINFO |
|  | “intergroup”) |  |  |
| S2 | DE"Treatment | Searchmodes- | Interface-EBSCOhost |
|  | Compliance"and(racial | Boolean/Phrase | SearchScreen-Advanced |
|  | orraceorethnic*or |  | Search |
|  | minorit*orcultur*) |  | Database-PsycINFO |
| S1 | (DE"CrossCultural | Searchmodes- | Interface-EBSCOhost |
|  | Psychology")or(DE | Boolean/Phrase | SearchScreen-Advanced |
|  | "CrossCultural |  | Search |
|  | Treatment")or(DE |  | Database-PsycINFO |
|  | "CrossCultural |  |  |
|  | Counseling")or(DE |  |  |
|  | "CrossCultural |  |  |
|  | Communication")or(DE |  |  |
|  | "CrossCultural |  |  |
|  | Differences")or(DE |  |  |
|  | "Acculturation")or(DE |  |  |
|  | "CultureChange")or(DE |  |  |
|  | "CulturalSensitivity") |  |  |
