# Supplementary material for: Cultural adaptations to augment health and mental health services: a systematic review
Source: BMC Health Serv Res. 2017 Jan 5;17:8. doi: 10.1186/s12913-016-1953-x (PMC5217593; doi:10.1186/s12913-016-1953-x)
Supplement: Additional file 4: — Grey Literature Sources. A list of sources used at the time of the grey literature search, with year searched if the link changed between 2012 and 2015. (DOCX 20 kb) [file 12913_2016_1953_MOESM4_ESM.docx]

Additional file 4

# Grey Literature Sources

| Source | Site |
| --- | --- |
| Agency for Healthcare Research and Quality, Minority Health, USA | http://www.ahrq.gov/research/minorix.htm |
| Campbell Collaboration, USA | http://www.campbellcollaboration.org/c2_systematic_reviews/index.php |
| Canadian Evaluation Society (2012) | http://www.evaluationcanada.ca/ |
| Canadian Institute for Health Information | http://www.cihi.ca |
| Center for Health Equity Research and Promotion, Veterans’ Affairs, USA | http://www.cherp.research.va.gov/http://www.hsrd.research.va.gov/publications/esp/healthcare-disparities.pdf |
| CENTRAL (Cochrane Central Registry of Controlled Trials), UK | http://onlinelibrary.wiley.com/o/cochrane/cochrane_clcentral_articles_fs.html |
| ClinicalTrials.gov | https://clinicaltrials.gov/ |
| Cochrane Database of Systematic Reviews, UK (2012) | http://www.thecochranelibrary.com/view/0/index.html |
| Cochrane Database of Systematic Reviews, UK (2015) | http://onlinelibrary.wiley.com/cochranelibrary/search |
| DARE, Database of Abstracts of Reviews of Effects, UK (2012) | http://onlinelibrary.wiley.com/o/cochrane/cochrane_search_fs.html?newSearch=true |
| DARE, Database of Abstracts of Reviews of Effects, UK (2015) | http://onlinelibrary.wiley.com/o/cochrane/cochrane_cldare_articles_fs.html |
| Greylit.org, The New York Academy of Medicine, Eliminating Health Disparities, USA | http://greylit.org/ |
| KaiserEDU.org, Frameworks for Addressing Racial Disparities, USA (2012) | http://www.kaiseredu.org/ |
| KaiserEDU.org, Frameworks for Addressing Racial Disparities, USA | http://kff.org/ |
| Mental Health Agency of Canada, CA | http://strategy.mentalhealthcommission.ca/strategy/reducing-disparities-and-addressing-diversity/ |
| National Center for Cultural Competence, USA | http://nccc.georgetown.edu/ |
| National Collaborating Centres (NCC) for Public Health, CA | http://www.nccph.ca/25/2010.ccnsp |
| National Institute for Health and Care Excellence (NICE), UK (2015) | http://www.nice.org.uk/ |
| National Library of Medicine (NLM Gateway), USA | http://gateway.nlm.nih.gov/ |
| NTIS, National Technical Information Service, Category: Health Care, USA (2012) | http://www.ntis.gov/search/index.aspx |
| NTIS, National Technical Information Service, Category: Health Care, USA (2015) | https://ntrl.ntis.gov/NTRL/login.xhtml |
| Office of Minority Health, U.S. Department of Health and Human Services, USA | http://minorityhealth.hhs.gov/ |
| PROSPERO, International Prospective Register of Systematic Reviews, UK | http://www.crd.york.ac.uk/prospero/ |
| Public Health Agency of Canada, CA | http://www.phac-aspc.gc.ca/index-eng.php |
| Public Health Agency of Canada Best Practices Portal, CA | http://cbpp-pcpe.phac-aspc.gc.ca/?s=+&post_type=interventions |
| Turning Research Into Practice (TRIP) Evidence Based Synopses, UK | https://www.tripdatabase.com/ |
| UK National Health Service, Library for Medicines (NeLM), UK | http://www.nelm.nhs.uk/en/ |

References Used to Identify Final Databases for Search

1. Canadian Agency for Drugs and Technologies in Health. Grey Matters. https://www.cadth.ca/resources/finding-evidence/grey-matters-practical-search-tool-evidence-based-medicine
2. Cochrane Guidelines and Library. http://www.thecochranelibrary.com
3. Giustini D. Finding the hard to finds: Searching for grey literature. 2012; http://blogs.ubc.ca/dean/files/2009/02/greylit_manual_may111.pdf
4. Grey Net International. Grey Source Index. http://www.greynet.org/greysourceindex.html
5. National Library of Medicine. National Information Center on Health Services Research and Health Care Technology. http://www.nlm.nih.gov/nichsrOntario Public Health Libraries Association.
6. Public health grey literature database: Overview. http://ophla.pbworks.com/w/page/35497970/Public%20Health%20Grey%20Literature%20Database%3A%20
7. Overview University of New Mexico Library. Gray Literature. http://hsc.unm.edu/library/subject/graylit.shtml
