# Supplementary material for: Cultural adaptations to augment health and mental health services: a systematic review
Source: BMC Health Serv Res. 2017 Jan 5;17:8. doi: 10.1186/s12913-016-1953-x (PMC5217593; doi:10.1186/s12913-016-1953-x)
Supplement: Additional file 5: — Detailed Table of Tested Adaptations and Outcomes by Report. A table sorting retained studies, their adaptations, and their outcome variables in accordance with the conceptual adaptation and outcome frameworks (Tables 3 and 4). (DOCX 83 kb) [file 12913_2016_1953_MOESM5_ESM.docx]

Additional file 5

Detailed Table of Tested Adaptations and Outcomes by Report

This table details how the retained studies, adaptations, and outcomes correspond to the conceptual frameworks outlined in Figures 2 and 3. It provides a brief synopsis of the intervention and outcomes with regard to target group and permits an in-depth view of how the intervention was adapted. Appendix E can be used in concert with Table 3, which provides a detailed summary of results from each study. This appendix may be used to identify studies with similar adaptations and/or outcomes, and Table 3 may be used to compare their results.

| **Report** | **Intervention and Cultural Adaptation(s) Tested in Isolation** | **Service Provider Behavioural Outcomes** | **Service Uptake (treatment) ^[[1]](#footnote-1)^** | **Service Uptake (prevention)** | **Service Recipient Awareness, Knowledge, Attitudes** | **Service Recipient Behavioral Outcomes** | **Indicators of Health Status** |
| --- | --- | --- | --- | --- | --- | --- | --- |
| **1. Primary Adapted Area: Consultation with the Community** | | | | | | | |
| There were no studies retained that used consultation with the community as the primary method of adaptation. | | | | | | | |
| **2. Primary Adapted Area: Changes in Structure and Process of Service Delivery** | | | | | | | |
| Ard et al., 2008 [47]  Final Report  *(Treatment focus)* | 20-week *racially matched* group weight-loss program^[[2]](#footnote-2)^ for African Americans.  **2.e.** **Change in manner of service delivery**  Featuring: Changes to group organization (racial match of recipients).  All group members were African American. |  | Program retention |  |  | Fruit and vegetable intake  % Intake of calories from fat  Fiber intake (g/day)  Physical activity (>180 min/week) | Weight loss |
| Gondolf, 2008 [41]  Final Report  (1 of 3)^[[3]](#footnote-3)^  *(Treatment focus)* | 16-week *racially matched* group standard counseling program for African American domestic-violence offenders, compared to a racially-mixed group standard counseling program.  **2.d. Changes to service provider/presenter**  Featuring: Racial matching.  Counselors were African American community members.  **2.e. Change in manner of service delivery**  Featuring: Changes to group organization (racial match of recipients).  All group members were African-American. |  | Program completion |  |  |  |  |
| Havranek et al., 2012 [52]  Final Report  *(Treatment focus)* | A *values-affirmation* exercise to reduce stereotype-threat and boost self-efficacy of African American clients during race-discordant client-provider communications.  **2.f. Provision of supplemental resources, services, or support**  Featuring: Other  Recipients were given a values-affirmation exercise prior to an appointment with their primary physician, in which they self-identified personal values or self-defining skills and discussed their importance. |  | Requested / provided more information (medical condition*^[[4]](#footnote-4)^, therapeutic regimen, lifestyle, requests for services) |  | Visit satisfaction (patient)  Trust in provider |  |  |
| Jandorf et al., 2013a [45]  Interim Findings^[[5]](#footnote-5)^  *(Preventive focus)* | (See Jandorf et al., 2013b).  *Peer-led* patient navigation for African Americans referred for colonoscopy. |  |  | Obtained colonoscopy | Trust in navigator  Patient satisfaction  Rating of message and source credibility |  |  |
| Jandorf et al., 2013b^[[6]](#footnote-6)^ [46]  Final Report  *(Preventive focus)* | *Peer-led* patient navigation for African Americans referred for colonoscopy.  **2.d. Changes to service provider/presenter**  Featuring: Provider matching (other).  Clients were assigned a local peer navigator to help prepare them for the experience who had previously undergone colonoscopy screening and was recruited from the local area.  **2.e. Change in manner of service delivery**  Peer navigators modeled effective coping with concerns about the exam (e.g., anxiety, fear and/or discomfort) by discussing strategies that helped them get through their own colonoscopies. |  |  | Obtained colonoscopy |  |  |  |
| Kalichman et al., 1993 [40]  Final Report  (1 of 3) ^[[7]](#footnote-7)^  *(Preventive focus)* | *Racial and gender matching* of presenter to audience in an HIV/AIDS educational video for African American women, compared to the same video with white presenters.  **2.d. Changes to service provider/presenter**  Featuring: Racial and gender matching.  Presenters in the racially/ gender matched video were African American women. |  |  | Obtained HIV test | Knowledge and attitudes (AIDS/HIV)  Rating of presenter (expertise/concern) | Bought/requested condoms*  Tried to use more condoms  Sought more info regarding AIDS/HIV |  |
| Mohan et al., 2014 [53]  Final Report  *(Treatment focus)* | *Simple, illustrated* medication management tool to improve medication understanding and adherence among Latinos with low-literacy.  **2.f. Provision of supplemental services, resources, or support**  Featuring: Other  Recipients were provided a medication-management tool which was translated, illustrated, and simplified. |  |  |  | Knowledge (medication indication*, strength*, units*, frequency*) | Medication regimen adherence |  |
| Skaer et al., 1996 [51]  Final Report  *(Preventive focus)* | *Targeted free* mammograms for Latina women with low-incomes.  **2.f. Provision of supplemental services, resources, or support**  Featuring: Funds for a service or resource.  Voucher for a free mammogram to be used within the next 30 days. |  |  | Obtained mammogram* |  |  |  |
| **3. Primary Adapted Area: Adaptation of Content** | | | | | | | |
| Holt et al., 2009 [65]  Final Report  *(Preventive focus)* | *Spiritually-based* prostate cancer education session for African American men.  **1.b. Community involvement**  The intervention was developed in partnership with African American focus groups and churches.  **3.b. Inclusion of cultural content**  Featuring: Cultural allusions, positive cultural beliefs and values.  Content included “Spiritually Based Health Education” & “Use of Scripture as a Motivator for Change”. Written materials included spiritual themes or scripture along with wellness and prostate information. |  |  | Read materials* | Knowledge (prostate cancer, risk factors, cancer screening)  Beliefs (prostate cancer)  Self-efficacy (prostate cancer screening, informed decision-making)  Barriers to screening |  |  |
| Holt et al., 2012a [60]  Interim Findings  *(Preventive focus)* | (See Holt et al., 2012b).  *Spiritually themed* colorectal cancer education session for African Americans. |  |  |  | Knowledge^[[8]](#footnote-8)^ (CRC^[[9]](#footnote-9)^)  Perceived benefits (CRC screening)  Perceived benefits (FOBT^[[10]](#footnote-10)^)  Perceived barriers (FOBT)  Perceived benefits (colonoscopy)  Perceived barriers (colonoscopy) |  |  |
| Holt et al., 2012b [61]  Final Report  *(Preventive focus)* | *Spiritually themed* colorectal cancer education session for African Americans.  **1.b. Community involvement**  The intervention was developed through an iterative process of testing and refinement in the African American community.  **3.b. Inclusion of cultural content**  Featuring: Cultural allusions, culturally relevant factual information, and positive/negative cultural beliefs and values.  The spiritually-based educational content incorporated scripture and religious themes into the intervention framework. |  |  | Obtained FOBT (lifetime, past year**^[[11]](#footnote-11)^)  Obtained flexible sigmoidoscopy (lifetime, past 5 years)  Obtained colonoscopy (lifetime, past 10 years)  Obtained barium enema (lifetime, past 5 years) | Perceived benefits (CRC screening)  Perceived benefits (FOBT)  Perceived barriers (FOBT)  Perceived benefits (colonoscopy)  Perceived barriers (colonoscopy) |  |  |
| Johnson et al., 2005 [37]  Final Report  *(Preventive focus)* | 8-session, 50 min. *multicultural* anti-smoking curriculum.  **1.b. Community involvement**  An advisory group of cultural experts was consulted, and lessons/activities were pilot tested by members of the target communities.  **3.b. Inclusion of cultural content**  Featuring: Graphics, cultural allusions, and positive cultural beliefs and values.  The curriculum included values from Latino and Pacific-Island cultures, e.g., the Latino value of familism (interdependence of family members) and the Pacific Islander value of filial piety (respect for ancestors) were incorporated into activities. Sessions also included images of multicultural characters, culturally-themed activities, and referenced the history of Pacific Rim nations. |  |  |  |  | Smoking by 8^th^ grade (lifetime)  Smoking by 8^th^ grade (past month) |  |
| Kreuter et al., 2003 [55]; Kreuter et al., 2004 [54]^[[12]](#footnote-12)^  Interim Findings  *(Preventive focus)* | (See Kreuter et al., 2005)  *Culturally tailored* cancer education magazines to increase mammography/fruit & vegetable intake among African Americans. |  |  | Attention (received materials, read materials)^[[13]](#footnote-13)^ |  |  |  |
| Kreuter et al., 2005 [56]  Final Report  *(Preventive focus)* | *Culturally tailored* cancer education magazines to increase mammography/fruit & vegetable intake among African Americans.  **1.b. Community involvement**  All elements of the tailored content were developed with extensive feedback from the St. Louis African American community.  **3.a. Level of personal specificity**  Featuring: Individualized content.  Magazines were individually tailored according to each person’s baseline measure on:  Religiosity (church attendance, prayer, participation in religious ceremonies, spirituality, beliefs about God as a causal agent’), collectivism (‘family or group, not the individual is the basic unit of society’; ‘cooperation, concern, responsibility for others, family security, respect for traditions and elders’), racial pride (interest and involvement in traditional practices, and holding positive attitudes about one’s race); and present/future time orientation (tendency to think and act according to consequences that are primarily immediate or more distal – future time).  **3.b. Inclusion of cultural content**  Featuring: Culturally relevant factual information, and positive cultural beliefs and values |  |  | Obtained mammogram |  | Fruit and vegetable intake |  |
| Nollen et al., 2007 [67]  Final Report  *(Treatment focus)* | Take-home video and reading materials targeted to African American smokers.  **3.b. Inclusion of cultural content**  Featuring: Graphics, culturally relevant factual information, and positive/negative cultural beliefs and values.  Cultural themes featured in the video include: Communalism, religion/spirituality, connections to ancestors and history, commitment to family, intuition, links between race-related stressors and smoking, principles of Kwanza, etc. Cultural content was also included in “Pathways to Freedom”; the guide features cultural imagery, and targets smoking patterns and preferences, and quit-barriers specific to African Americans. |  | Used materials (guide* and video) |  | Perceived benefit (helpful in trying to quit: guide and video)  Readiness to quit (stages of change) | Smoking abstinence (past week)  Used quitting aids (nicotine patch)  Reduction in cigarettes |  |
| Resnicow et al., 2009 [57]  Final Report  *(Treatment focus)* | *Culturally tailored* fruit & vegetable promotional materials for African Americans.  **1.b. Community involvement**  A sample of participants responded to surveys, and answers were used to develop the materials later provided to recipients.  **3.a. Level of personal specificity**  Featuring: Individualized content.  Materials were tailored to recipients’ level of acculturation.  Text and images were tailored toward one of 16 types of ethnic identity for African Americans. The 16 groups were constructed from 6 subscales: Afrocentric, Black American, Bicultural, Multicultural, Racial Salience, and Cultural Mistrust. Tailored content included images and messages.  **3.b. Inclusion of cultural content**  Featuring: Graphics, culturally relevant factual information, and positive/negative cultural beliefs and values. |  | Read materials |  |  | Fruit and vegetable intake |  |
| Sanders Thompson et al., 2010 [64]  Final Report  *(Preventive focus)* | *Culturally tailored* colorectal cancer risk-reduction materials for African Americans.  **3.b.** **Inclusion of cultural content**  Featuring: Graphics, culturally relevant factual information, and positive/negative cultural beliefs and values.  Socio-cultural messages focused on ideals of collectivism and ethnic identity that cited family and community benefits, and countered issues of mistrust and the desire for privacy by emphasizing how these undermined health. Publications included localized photos to heighten relevance, and photos depicting church, family and community scenes. |  |  | Engagement (materials) | Affective reactions to publications (interest, motivation)  Cognitive processing (impact, ability to remember, use of information)  Ease of understanding  Intent to obtain colorectal cancer screening |  |  |
| Shoptaw et al., 2005 [72]  Final Report  *(Treatment focus)* | *Culturally tailored* cognitive behavioral therapy with reference to *cultural norms and values* of urban gay and bisexual men and emphasis on reduction of HIV-related sexual behaviors.  **3.b. Inclusion of cultural content**  Featuring: Cultural allusions and positive/negative cultural beliefs and values.  All topics used gay referents. For example, a session on identification of triggers to relapse provides standard CBT^[[14]](#footnote-14)^ information, but identifies gay cultural events (e.g., circuit parties) and environments (e.g., sex clubs) frequented by methamphetamine-using GBM^[[15]](#footnote-15)^. |  | Program retention |  |  | Substance use (meth)*^[[16]](#footnote-16)^  Unprotected sex | Addiction severity index |
| Webb, 2009 [69]  Final Report  *(Treatment focus)* | *Culturally targeted* written materials for smoking cessation among African Americans.  **3.b. Inclusion of cultural content**  Featuring: Graphics, cultural allusions, culturally relevant factual information, and positive/negative cultural beliefs and values.  Used “Pathways To Freedom”. Aspects of cultural content included: African American values (religion, collectivism), history, statistics, motivation to mobilize the African American community against the tobacco industry, etc. Pictures were exclusively African American, as were testimonials. Names were stereotypically African American, and colours were Pan African. |  | Used materials (read, used, saved) |  | Attitudes toward materials (captured attention*, encouraging*, found helpful*, trustworthy**^[[17]](#footnote-17)^, understandable*, satisfaction: content*)  Readiness to quit (Contemplation Ladder**) | Smoking abstinence / reduction (past day, past week)  Quit attempts (#)** |  |
| Webb et al., 2010 [63]  Final Report  *(Treatment focus)* | *Culturally targeted* written materials and program for smoking cessation among African Americans.  **3.b. Inclusion of cultural content**  Featuring: Graphics, cultural allusions, culturally relevant factual information, and positive/negative cultural values and beliefs.    The culturally specific interventions included socio-cultural, historical, environmental, and psychological factors of the target group, discussion of the history of slavery and smoking, targeted tobacco advertising, the prevalence of smoking-attributable deaths among African Americans, the health consequences of smoking for African Americans, and factors contributing to health disparities. Used “Pathways To Freedom” and included images of famous African Americans who died from smoking-related diseases (e.g., Sammy Davis Jr., Nat King Cole). |  |  |  | Perceived risk (smoking)*  Perceived cultural risk (smoking, as compared to whites)*  Knowledge (smoking)  Readiness to quit (Contemplation Ladder, intention to quit questionnaire*) |  |  |
| **Packages of Adaptations Including both Content and Structural Changes** | | | | | | | |
| Burrow-Sanchez & Wrona, 2012 [70]  Pilot Study  *(Treatment focus)* | (See Burrow-Sanchez et al., 2015)^[[18]](#footnote-18)^  *Culturally tailored* cognitive behavioral therapy for Latino adolescents with substance use disorders. |  | Program retention |  | Program satisfaction (parent)*  Program satisfaction (adolescent) | Illicit drug use (past 90 days) |  |
| Burrow-Sanchez et al., 2015 [71]  Final Report  *(Treatment focus)* | *Culturally tailored* cognitive behavioral therapy for Latino adolescents with substance use disorders.  **1.b. Community involvement**  The intervention was developed with input from focus groups involving stakeholders in the Latino community.  **2.e. Change in manner of service delivery**  Treatment delivery for parents was modified by holding a Family Introduction Meeting immediately prior to the beginning of the first group and promoting regular (i.e., every third session) phone and mail contact between the therapist and parents.  **3.b. Inclusion of cultural content**  Featuring: Cultural allusions, and positive/negative cultural beliefs and values.  Treatment was revised to increase cultural relevance by including Spanish names in examples, role-plays relevant for Latino adolescents (e.g., problem solving in the context of a racist environment) and opportunities to discuss frequently encountered stressors (e.g., translating for a parent, dealing with discrimination). |  |  |  |  | Illicit drug use (past 90 days) |  |
| Chiang & Sun, 2009 [73]  Final Report  *(Treatment focus)* | 8-week *culturally tailored* walking program for Chinese Americans.  **2.e. Change in manner of service delivery**  Family members’ involvement was sought by asking them to consent to the recipient’s participation in the program, thereby demonstrating their approval.  **3.b. Inclusion of cultural content**  Featuring: Positive cultural beliefs and values.  Intervention emphasized Chinese cultural values of authority, familial involvement, harmony, and balance. |  |  |  |  |  | Blood pressure  Walking endurance |
| Fitzgibbon et al., 2005 [44]  Final Report  *(Treatment focus)* | *Faith-based* 12-week weight-loss program for African Americans.  **2.d. Changes to service provider/presenter**  Featuring: Cultural matching.  The presenter was experienced with health risk reduction in minorities, and had a thorough knowledge of the Bible and scripture.  **3.b. Inclusion of cultural content**  Featuring: Cultural allusions, and positive cultural beliefs and values.  Each weekly group session incorporated scripture into the program content. |  | Program retention |  |  | Physical activity / energy expenditure (per day)  Dietary fat consumption | Weight change  BMI^[[19]](#footnote-19)^ change |
| Gondolf, 2008 [41]  Final Report  (2 of 3) ^[[20]](#footnote-20)^  *(Treatment focus)* | 16-week *culturally-tailored*, racially-matched group counseling program for African American domestic-violence offenders with an ethnically matched provider, compared to a racially matched provider and standard curriculum.  **2.d. Changes to service provider/presenter**  Featuring: Cultural matching.  Counselor was a community member involved in social services who lived in a predominantly African American neighborhood, and had strong community ties.  **3.b.** **Inclusion of cultural content**  Featuring: Positive/negative cultural beliefs and values.  Cultural topics and themes included African-American men’s perceptions of the police, relationships with women, sense of African-American manhood, reactions to discrimination and prejudice, and support in the African-American community. Other topics included being oppressed and being the oppressor, finding peace when you feel powerless, and exploring the roots of violence in one’s life. Positive aspects of African-American culture were also incorporated including sense of brotherhood, communal spirit, intuitive insight, poetic expression, spirituality, and ritual. |  | Program completion |  |  |  |  |
| Gondolf, 2008 [41]  Final Report  (3 of 3)  *(Treatment focus)* | 16-week *culturally-tailored and ethnically-matched* group counseling program for African American domestic-violence offenders, compared to a multi-race group and standard curriculum with white providers.  **2.d. Changes to service provider/presenter**  Featuring: Racial and cultural matching.  Counselors were African American community members.  Counselor had experience in community-based social services, and lived in a predominantly African American neighborhood with strong identification with its cultural and social issues.  **2.e. Change in manner of service delivery**  Featuring: Changes to group organization (racial match of recipients).  All group members were African-American.  **3.b.** **Inclusion of cultural content**  Featuring: Positive/negative cultural beliefs and values.  Cultural topics and themes included African-American men’s perceptions of the police, relationships with women, sense of African-American manhood, reactions to discrimination and prejudice, and support in the African-American community. Other topics included being oppressed and being the oppressor, finding peace when you feel powerless, and exploring the roots of violence in one’s life. Positive aspects of African-American culture were also incorporated including sense of brotherhood, communal spirit, intuitive insight, poetic expression, spirituality, and ritual. |  | Program completion |  |  |  |  |
| Halbert et al., 2010 [62]  Final Report  *(Preventive focus)* | *Culturally tailored* genetic counseling for African American women.  **2.e. Change in manner of service delivery**  The session used structured probes to elicit discussion of women’s beliefs and values (religion, temporal orientation, communalism), and made use of a genogram.  **3.b. Inclusion of cultural content**  Featuring: Positive cultural beliefs and values.  In one-time individual genetic counseling sessions, discussion of cultural values and beliefs was related to health care decision-making (e.g., spirituality and religion, temporal orientation, and communalism). |  |  | Obtained genetic screening  Program completion | Perceived risk (breast cancer) |  |  |
| Huey & Pan, 2006 [58]  Pilot Study  *(Treatment focus)* | (See Pan et al., 2011) ^[[21]](#footnote-21)^  *Culturally tailored* single-session exposure treatment for Asian Americans with phobias. |  |  |  |  |  | Reduction of phobic symptoms  Distress  Catastrophic thinking*^[[22]](#footnote-22)^  General fear |
| Hwang et al., 2015 [74]  Final Report  *(Treatment focus)* | *Culturally adapted* cognitive behavioral therapy for Latinos with depression.  **1.b. Community involvement**  Treatment manual was developed in consultation with Asian American mental health clinics, traditional Chinese medicine practitioners, and Chinese spiritual leaders of Buddhism/Taoism.  **2.e. Change in manner of service delivery**  Therapists spent more time orienting clients to therapy, building rapport, and educating them about expected progress and the course of therapy. The therapeutic process was normalized, and all statements directed to clients were framed in a way that accounted for their personal sense of spirituality, familism, and of being stereotyped. Therapists engaged in the cultural rules of etiquette and practiced competence in Asian communicative styles.  **3.b. Inclusion of cultural content**  Featuring: Graphics, cultural allusions, and positive/negative cultural beliefs and values.  Materials were culturally salient and visually appealing. Tasks and topics made reference to Asian beliefs (e.g., bridging Qi, balancing energy) and used relevant metaphors to facilitate understanding. Positive cultural beliefs were elicited as motivating factors for change. |  | Program retention |  |  |  | Severity of depressive symptoms*^[[23]](#footnote-23)^ |
| Kalichman et al., 1993 [40]  Final Report  (2 of 3) ^[[24]](#footnote-24)^  *(Preventive focus)* | *Culturally tailored content* in an AIDS/HIV educational video for African American women, compared to a racial/ gender-matched video with standard content.  **1.b. Community involvement**  A focus group of African-American women identified culturally and personally relevant video themes.  **2.e. Change in manner of service delivery**  Presenters in the video dressed more casually and spoke less formally.  **3.b. Inclusion of cultural content**  Featuring: Graphics, culturally relevant factual information, and positive cultural values and beliefs.  The video stressed culturally relevant values, such as cultural pride, concern for the community, and familial responsibility. Footage of African American families was spliced into the film. |  |  | Obtained HIV test* | Knowledge (AIDS/HIV)  Sensitization to AIDS/HIV    Rating of presenter (expertise/concern*) | Bought/requested condoms  Sought more info regarding AIDS/HIV |  |
| Kalichman et al., 1993 [40]  Final Report  (3 of 3)  *(Preventive focus)* | *Culturally tailored content* and *racial/gender matching* in an AIDS/HIV educational video for African American women, compared to a standard video with white presenters.  **1.b. Community involvement**  A focus group of African-American women identified culturally and personally relevant video themes.  **2.d. Changes to service provider/presenter**  Featuring: Racial and gender matching.  Presenters in the racially/ gender matched video were African American women.  **2.e. Change in manner of service delivery**  Presenters in the video dressed more casually and spoke less formally.  **3.b. Inclusion of cultural content**  Featuring: Graphics, culturally relevant factual information, and positive cultural values and beliefs.  The video stressed culturally relevant values, such as cultural pride, concern for the community, and familial responsibility. Footage of African American families was spliced into the film. |  |  | Obtained HIV test* | Knowledge (AIDS/HIV)  Sensitization to AIDS/HIV    Rating of presenter (expertise/concern*) | Bought/requested* condoms  Sought more info regarding AIDS/HIV |  |
| La Roche et al., 2006 [38]  Pilot Study  *(Preventive focus)* | *Culturally adapted* educational asthma management program for African American and Latino families.  **2.e. Change in manner of service delivery**  Providers encouraged collaboration among families present and use of cultural resources in asthma management. They elicited beliefs and stories about asthma-related experiences, and families enrolled together remained in contact between testing points.  **3.b. Inclusion of cultural content**  Featuring: Cultural allusions, culturally-relevant factual information, and positive cultural beliefs and values.  Asthma symptoms and experiences were framed in historical and sociocultural contexts. Culturally relevant risk factors and resources were discussed. |  |  |  | Asthma knowledge (parents, children)*  Asthma skills (parents, children) |  | # Asthma-induced emergency room visits (past year)* |
| Lee et al., 2013 [48]  Final Report  *(Treatment focus)* | *Culturally tailored* single-session motivational interviewing for Latinos with alcohol-induced behavioral problems.  **1.b. Community Involvement**  The culturally adapted intervention was developed and piloted with members of the Latino community.    **2.e. Change in manner of service delivery**  Therapists were trained to build rapport and collaborate with clients by eliciting and discussing participant ideas about cultural/social influences on their drinking and cultural motivations to change.  **2.f. Provision of supplemental resources, services, or support**  Featuring: Supplemental services  Child care and transportation were provided when needed, and sessions were conducted after work hours and on the weekends to accommodate work schedules.  **3.b. Inclusion of cultural content**  Featuring: Culturally relevant factual information and positive/negative cultural beliefs and values.  The main theme was to address the social context of drinking and acculturation stressors that influence drinking behavior, including the effects of low-status employment and the importance of family. |  | Engagement (treatment) |  | Program satisfaction | Alcohol use (drinking days per month)  Heavy alcohol use (heavy drinking days per month) | Severity of alcohol problems*^[[25]](#footnote-25)^ |
| McCabe & Yeh, 2009 [49]  Interim Findings  *(Treatment focus)* | (See McCabe et al., 2012)  *Culturally tailored* Parent-Child Interaction Therapy for Mexican American families with children who have behavior problems. |  | Program attendance / dropout |  | Program satisfaction | Quality of parent-child interactions ^[[26]](#footnote-26)^  Positive parenting behavior | Child ADHD^[[27]](#footnote-27)^ symptoms  Child conduct disorder symptoms  Child ODD^[[28]](#footnote-28)^ symptoms  Parental stress  Problematic child behavior |
| McCabe et al., 2012 [50]  Final Report  *(Treatment focus)* | *Culturally tailored* Parent-Child Interaction Therapy for Mexican American families with children who have behavior problems.  **1.b. Community involvement**  The intervention was developed with the influence of cultural experts and Mexican-American families.  **2.e. Change in manner of service delivery**  Treatment was framed as an education/skill building program. Orientation to therapy was also increased. Session time was increased for rapport building. The provider engaged families in activities designed to promote program retention.  **2.f. Provision of supplemental services, resources, or support**  Featuring: Translated materials.  Written hand-outs were translated and simplified.  **3.a. Level of personal specificity**  Featuring: Individualized content.  Each family had a detailed needs assessment of cultural values, such as beliefs regarding the cause of children’s behaviour, beliefs about discipline, beliefs about the roles family members should play, etc.  **3.b. Inclusion of cultural content**  Featuring: Graphics, and positive/negative cultural values and beliefs.  Written handouts had added representations of Mexican American families. |  |  |  |  |  | Child ADHD symptoms  Child conduct disorder symptoms  Child ODD symptoms  Parental stress  Problematic child behavior  Parental locus of control^[[29]](#footnote-29)^ |
| Newton & Perri, 2004 [39]  Final Report  *(Treatment focus)* | 10-session group-exercise promotion program and written materials for African Americans.  **2.a. Change in geography/location**  Sessions were held at a site located within the African American community.  **2.d. Changes to service provider/presenter**  Featuring: Racial matching.  Sessions were led by African-American counselors.  **2.e. Change in manner of service delivery**  Featuring: Changes to group organization (racial match of recipients).  All group members were African-American.  **3.b.** **Inclusion of cultural content**  Featuring: Graphics, cultural allusions, and positive cultural beliefs and values.  Materials addressed sociocultural concerns of African Americans and related them to exercise, including cultural history, worldview, and belief system. Graphics included visual depictions of African Americans, and print materials adorned with Kente cloth patterns. |  | Program adherence |  | Self-efficacy (for exercise behavior)  Program satisfaction (leader appreciation)*^[[30]](#footnote-30)^ | Physical activity | Cardio-respiratory fitness (VO_2_ max) |
| Orleans et al., 1998 [68]  Final Report  *(Treatment focus)* | *Culturally targeted* stop-smoking counseling session and written materials for African Americans.  **2.e. Change in manner of service delivery**  Phone counselors adopted an interactive communication style to facilitate commitment to a quit-plan.  **3.b. Inclusion of cultural content**  Featuring: Graphics, culturally relevant factual information, and positive/negative cultural belief and values.  Quit smoking strategies were geared to the modal smoking patterns of African American smokers, and addressed specific obstacles that African American smokers face in their attempts to stop smoking. Cultural content was also included in “Pathways to Freedom”: the guide features cultural imagery, and targets quit-barriers specific to African Americans. |  | Read materials |  | Intent to quit (stages of change) (6 and 12*  months)  Satisfaction (found guide helpful, suitable for family**) | Quit attempts (#) (6* and 12 months)  Smoking abstinence (past week) (6 and 12*  months)  Reduction in cigarettes (6 months)*  Set quit date (6 months)*  Switched to lower nicotine brand (6 months)*  Used quitting aids (nicotine patch/gum) (12 months) |  |
| Pan et al., 2011 [59]  Final Report  *(Treatment focus)* | [See also Huey & Pan, 2006] ^[[31]](#footnote-31)^  *Culturally tailored* single-session exposure treatment for Asian Americans with phobias.  **1.b. Community involvement**  Adaptations were developed to be consistent with the recommendations of Asian-American experts, then pilot tested and refined with members of the Asian-American community.  **2.e. Change in manner of service delivery**  The therapist adopted an authoritative style, using directive statements rather than passive queries.  **3.b. Inclusion of cultural content**  Featuring: Individualization to recipient’s level of acculturation.    Incorporated seven empirically-supported strategies to improve phobic outcomes for Asian American recipients: They assessed and addressed acculturation status, addressed clients’ explanatory models of the target problems, targeted their normalization of the experience, emphasized confidentiality, emphasized emotional control, emphasized the therapist’s authority, and provided psychoeducation. |  |  |  |  |  | Behavioural assessment of avoidance and anxiety  Subjective distress  Clinician rating of client’s fear*  General fear  Catastrophic thinking  DSM-IV-TR^[[32]](#footnote-32)^ phobic symptoms |
| Unger et al., 2013 [66]  Final Report  *(Treatment focus)* | *Culturally tailored fotonovela* to increase depression knowledge and reduce stigma among Latinos.  **1.b. Community involvement**  The fotonovela was developed and piloted in focus groups with the Latino community. The script was written by a local Latino author, and materials were translated by Latino members of the research team.  **2.e. Change in manner of service delivery**  Information was delivered in a culturally-relevant, storytelling format, recounting the decision of a Hispanic wife and mother to receive counselling and medication for her depression.  **3.b. Inclusion of cultural content**  Featuring: Graphics, cultural allusions, and positive/negative cultural beliefs and values.  Fotonovela made reference to Latino characters with Latino names, and referred to cultural beliefs such as familism or stigma surrounding mental health. |  |  |  | Knowledge (depression)*^[[33]](#footnote-33)^  Stigma (antidepressants, mental health care)*  Self-efficacy (to recognize depression)  Willingness to seek help |  |  |
| Wang et al., 2012a [43]  Interim Findings  *(Preventive focus)* | (See Wang et al., 2012b)  *Culturally targeted,* mailed promotional video to increase mammography among Chinese-American immigrants. |  |  |  | Intent to obtain mammogram^[[34]](#footnote-34)^  Eastern cultural views of healthcare  Knowledge (breast cancer)  Perceived risk (breast cancer)    Perceived benefits (mammography)  Perceived barriers (mammography) |  |  |
| Wang et al. 2012b [42]  Final Report  *(Preventive focus)* | *Culturally targeted,* mailed promotional video to increase mammography among Chinese-American immigrants.  **1.b.Community involvement**  Focus groups were conducted with Chinese women to identify preferred video content and format.  **2.d. Changes to service provider/presenter**  Featuring: Racial and language matching.  The cultural video featured Chinese actors and presenters to deliver educational information. It was filmed in Mandarin and dubbed in Cantonese with both Chinese and English subtitles.  **3.b. Inclusion of Cultural Content**  Featuring: Graphics, cultural allusions, and positive/negative cultural beliefs and values.  The video is designed to debunk Chinese women’s culturally based beliefs about breast cancer and attitudes towards regular mammograms, and featured cultural referents (e.g., cultural décor, music), statistics, and Chinese characters. |  |  | Obtained mammogram |  |  |  |

1. Note that “Service Uptake” has been split into two distinct categories to better identify outcomes. [↑](#footnote-ref-1)
2. *Italicized* text indicates the *value* or *cultural facet* included in the intervention. Underlined text represents the intervention itself. Red text denotes the comparison group when a study tests more than one manipulation and multiple comparisons can be made. [↑](#footnote-ref-2)
3. Gondolf (2008) [41] allowed for three distinct comparisons to be made, all of which are detailed in this table. The remaining two comparisons are located below, under the heading “Packages of Adaptations Including both Content and Structural Changes”. [↑](#footnote-ref-3)
4. An asterisk (*) indicates that a significant effect was found at follow-up. Significance is defined by the author’s criteria in the original report. See Table 4 for a detailed list of study findings. [↑](#footnote-ref-4)
5. Related reports in sequence which are part of the same study are delineated by a dashed line instead of a double solid line. [↑](#footnote-ref-5)
6. Jandorf et al. (2013a) [45] is an interim report from the same study as Jandorf et al. (2013b) [46]. The outcomes “trust in navigator”, “program satisfaction”, and “rating of message and source credibility” are not assessed in the final report, Jandorf et al. (2013b) [46]. [↑](#footnote-ref-6)
7. Kalichman et al. (1993) [40] allow for three distinct comparisons to be made, all of which are detailed in this table. The remaining two comparisons are located below the heading “Packages of Adaptations Including both Content and Structural Changes”. The asterisk in this instance indicates significance for this specific comparison. [↑](#footnote-ref-7)
8. Holt et al. (2012a) [60] is an interim report from the same study as Holt et al. (2012b) [61]. The outcome “CRC knowledge” was not assessed in the final report, Holt et al. (2012b) [61]. [↑](#footnote-ref-8)
9. Colorectal Cancer (CRC). [↑](#footnote-ref-9)
10. Fecal Occult Blood Test (FOBT). [↑](#footnote-ref-10)
11. **Favors the Standard condition. [↑](#footnote-ref-11)
12. Kreuter et al. (2003) [55] and Kreuter et al. (2004) [54] have been combined in this table because both reports analyze the same data collected from one sample at the same points in time. [↑](#footnote-ref-12)
13. Kreuter et al. (2003, 2004) [55, 54] are interim reports from the same study as Kreuter et al. (2005) [56]. The outcome “Attention” was not assessed in the final report, Kreuter et al. (2005) [56]. [↑](#footnote-ref-13)
14. Cognitive Behavioral Therapy (CBT). [↑](#footnote-ref-14)
15. Gay or Bisexual Men (GBM). [↑](#footnote-ref-15)
16. * Significant only for treatment effectiveness score at end of treatment. [↑](#footnote-ref-16)
17. ** Favors the Standard group. [↑](#footnote-ref-17)
18. Burrow-Sanchez et al. (2012) [71] is a completed pilot study. The data reported in this study come from a unique sample and are distinct from the data reported in Burrow-Sanchez et al. (2015) [70], although the two reports represent the same design. [↑](#footnote-ref-18)
19. Body Mass Index (BMI). [↑](#footnote-ref-19)
20. Gondolf (2008) [41] allows for three distinct comparisons, all of which are detailed in this table. Two comparisons are located here, beneath the heading “Packages of Adaptations Including both Content and Structural Changes”. The remaining comparison can be found above, under the heading “Changes in Structure and Process of Service Delivery”. [↑](#footnote-ref-20)
21. Huey & Pan (2006) [58] is a completed pilot study. The data were later folded into the analysis of Pan et al. (2011) [59]. The outcomes analyzed and reported are the same in each report. [↑](#footnote-ref-21)
22. CA group outperformed STD group on reducing catastrophic thinking in this pilot, but this finding was not present within the larger study, Pan et al. (2011) [59]. [↑](#footnote-ref-22)
23. The decrease in depression scores in the Culturally Adapted group was significantly greater than that in the Standard group, though groups did not significantly differ in post-test depression scores. [↑](#footnote-ref-23)
24. Kalichman et al. (1993) [40] allows for three distinct comparisons, all of which are detailed in this table. Two comparisons are located here, below the heading “Packages of Adaptations Including both Content and Structural Changes”. The remaining can be found above, under the heading “Changes in Structure and Process of Service Delivery”. [↑](#footnote-ref-24)
25. * Significant for the Impulse subscale of the DrInC scale. [↑](#footnote-ref-25)
26. McCabe & Yeh (2009) [49] is an interim report from the same study as McCabe et al. (2012) [50]. The outcomes “program attendance/dropout”, “program satisfaction”, “quality of parent-child interactions” and “positive parenting behavior” are not assessed in the final report, McCabe et al. (2012) [50]. [↑](#footnote-ref-26)
27. Attention Deficit Hyperactivity Disorder (ADHD). [↑](#footnote-ref-27)
28. Oppositional Defiant Disorder (ODD). [↑](#footnote-ref-28)
29. Only the final report, McCabe et al. (2012) [50], reports on the outcome “parental locus of control”. [↑](#footnote-ref-29)
30. * Significant for rating presenter as expressing appreciation. [↑](#footnote-ref-30)
31. Pan et al. (2011) [59] is the wider report on the same intervention as Huey & Pan (2006) [58]. The data from Huey & Pan (2006) [58] have been folded into Pan et al. (2011) [59]. The outcomes analyzed and reported are the same in each report. [↑](#footnote-ref-31)
32. Diagnostic and Statistical Manual of Mental Disorders, 4th Edition, Text Revision (DSM-IV-TR). [↑](#footnote-ref-32)
33. Outcomes for Unger et al. (2013) [66] are reported from post-test, as the follow-up data was confounded when participants in either group exchanged reading materials after the post-test measure. [↑](#footnote-ref-33)
34. Wang et al. (2012a) [43] is an interim report from the same study as Wang et al. (2012b) [42]. The outcomes “intent to obtain a mammogram”, “eastern cultural view of healthcare”, “knowledge”, “perceived risk”, “perceived benefits”, and “perceived barriers” are not assessed between target groups in the final report, Wang et al. (2012b) [42]. [↑](#footnote-ref-34)
