# Supplementary material for: Cultural adaptations to augment health and mental health services: a systematic review
Source: BMC Health Serv Res. 2017 Jan 5;17:8. doi: 10.1186/s12913-016-1953-x (PMC5217593; doi:10.1186/s12913-016-1953-x)
Supplement: Additional file 6: — One Example of a Conceptual Hierarchy of Client Engagement. An example of another potential dimension by which adaptations could be evaluated. (DOCX 20 kb) [file 12913_2016_1953_MOESM6_ESM.docx]

Additional file 6

One Example of a Conceptual Hierarchy of Client Engagement^[[1]](#footnote-1)^

| Method | Level of Engagement | Cultural Adaptation |
| --- | --- | --- |
| Space | Low  ↕  High | ► Located where other service is provided  ► Located within cultural community  ► Service delivered at home |
| *Surface* *structure* of content^[[2]](#footnote-2)^ | Low  ↕  High | ► Visual depictions – colors, layout, symbols, etc.  ► Visual depictions - individuals  ► Evidence-based data on issue in relation to cultural group  ► Cultural content/messages  ► Cultural group tailoring of materials (within racial/ethnic group)  ► Individual cultural tailoring |
| *Deep structure* of content^2^ | Low  ↕  High | ► Evidence-based data on issue in relation to cultural group  ► Cultural content – for purpose of relating to context/identification with group  ► Cultural content – Negative sociocultural experiences as relational or motivational  ► Cultural content – positive cultural values as motivating factors  ► Culturally tailored curricular adaptations following cultural assessment |
| Manner of service delivery | Low  ↕  High | ► Within group racial match  ► Racial match of provider  ► Racial and cultural match of provider  ► Discussion of cultural issues (passive – responses)  ► Discussion of cultural issues (active – probes)  ► Evidence-based interaction style  ► Individually tailored interaction style following cultural/individualized assessment |

1. Adaptations vary in their degree of engagement of members of the target population. Those areas of adaptation depicted are a few of the many examples possible given the adaptation framework of Figure 2. [↑](#footnote-ref-1)
2. Surface and deep structures were described by Resnicow K, Soler R, Braithwaite RL, Ahluwalia JS, Butler J. Cultural sensitivity in substance use prevention. Journal of community psychology. 2000 May 1;28(3):271-90. [↑](#footnote-ref-2)
