# Supplementary material for: Cultural adaptations to augment health and mental health services: a systematic review
Source: BMC Health Serv Res. 2017 Jan 5;17:8. doi: 10.1186/s12913-016-1953-x (PMC5217593; doi:10.1186/s12913-016-1953-x)
Supplement: Additional file 7: — Forest Plot - Intervention Effects by Number of Adapted Elements. A forest plot illustrating treatment efficacy sorted by number of adapted elements. (DOCX 84 kb) [file 12913_2016_1953_MOESM7_ESM.docx]

Forest Plot - Intervention Effects by Number of Adapted Elements

This forest plot depicts the relative mean differences for studies reporting sufficient data for testing. This plot addresses Hasnain et al.’s^[[1]](#endnote-1)^ question about the potential influence of numerous adaptations combined. The forest plot used “inverse variance” and “random effect” estimates in Review Manager 5.3.


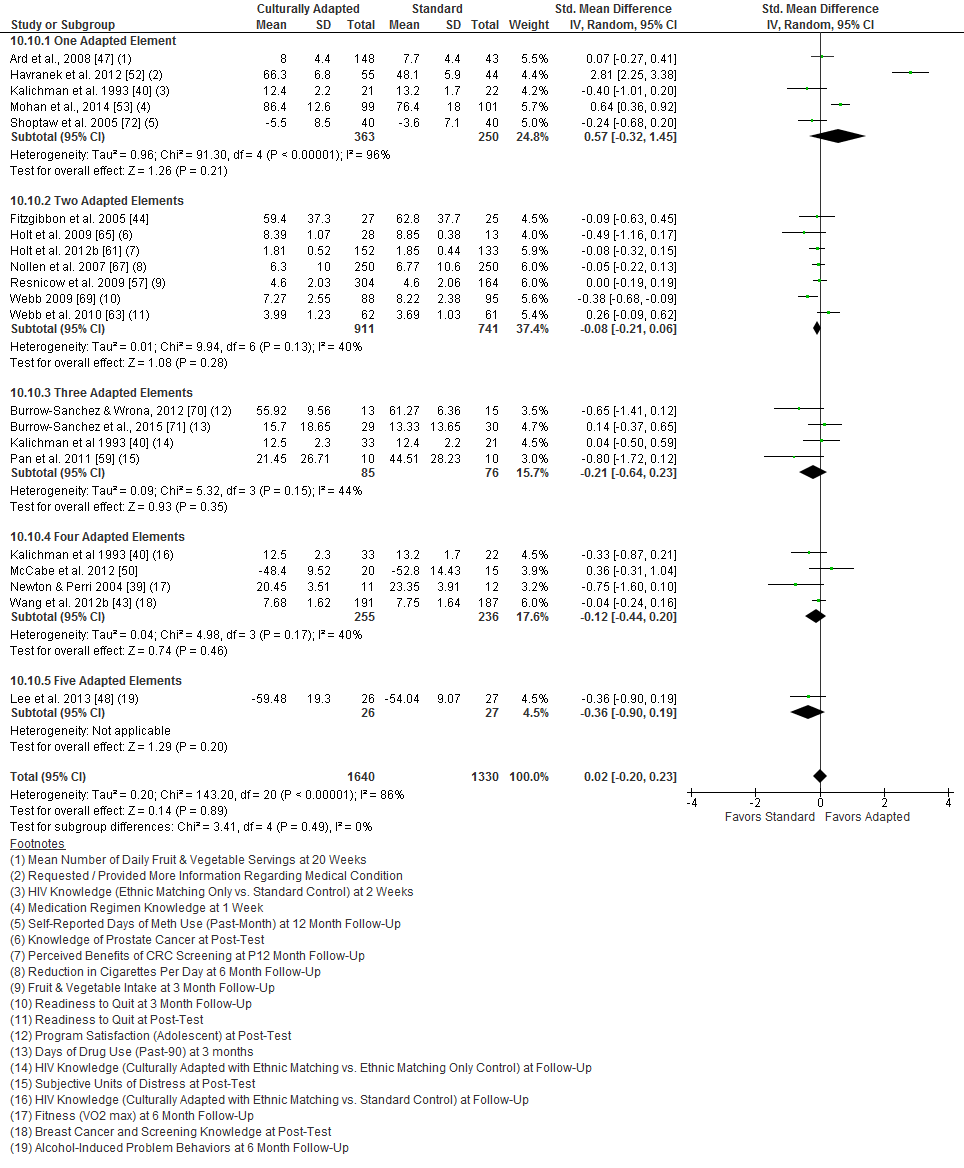


1. Hasnain R, Kondratowicz DM, Portillo N, Borokhovski E, Balcazar F, Johnson T, et al. The use of culturally adapted competency interventions to improve rehabilitation service outcomes for culturally diverse individuals with disabilities. Submitted to the Campbell Collaboration, Education Coordinating Group. 2009; http://www.ncddr.org/partners/subgroup/resources/hasnain_competency_interventions_review_2010.pdf [↑](#endnote-ref-1)
